# Transcriptome profiling and proteomic validation reveals targets of the androgen receptor signaling in the BT-474 breast cancer cell line

**DOI:** 10.1186/s12014-022-09352-2

**Published:** 2022-05-14

**Authors:** Stella K. Vasiliou, Panagiota S. Filippou, Sergi Clotet-Freixas, Antoninus Soosaipillai, Ihor Batruch, Foivos Viktor Tsianos, Ana Konvalinka, Eleftherios P. Diamandis

**Affiliations:** 1 Department of Laboratory Medicine and Pathobiology, University of Toronto, Toronto, ON Canada; 2 Department of Pathology and Laboratory Medicine, Mount Sinai Hospital, Toronto, ON Canada; 3 Department of Clinical Biochemistry, University Health Network, Toronto, ON Canada; 4 Toronto General Hospital Research Institute, University Health Network, Toronto, Canada; 5 Soham and Shaila Ajmera Family Transplant Centre, University Health Network, Toronto, Canada; 6 Canadian Donation and Transplantation Research Program, Edmonton, Canada; 7 Department of Medicine, Division of Nephrology, University Health Network, Toronto, Canada; 8 Institute of Medical Science, University of Toronto, Toronto, Canada; 9 Present Address: School of Health & Life Sciences, Teesside University, Middlesbrough, TS1 3BX UK; 10 Present Address: National Horizons Centre, Teesside University, Darlington, DL1 1HG UK; 11 Mount Sinai Hospital, Joseph & Wolf Lebovic Ctr., 60 Murray St. [Box 32]; Flοοr 6- Rοοm L6-201, Toronto, ON M5T 3L9 Canada

**Keywords:** Breast cancer, Transcriptome, Androgen receptor, Sex hormones, RNA sequencing, BT-474, Metabolism

## Abstract

**Background:**

Accumulating evidence suggests that the androgen receptor (AR) and its endogenous ligands influence disease progression in breast cancer (BCa). However, AR-mediated changes in BCa differ among the various BCa subtypes according to their hormone receptor profile [i.e., presence/absence of estrogen receptor (ER), progesterone receptor (PR) and human epidermal growth factor receptor 2, (HER2)]. Thus, we explored the androgen-regulated transcriptomic changes in the ER^+^PR^+^HER2^+^ BCa cell line, BT-474, and compared them with PR-mediated changes.

**Methods:**

We performed RNA sequencing analysis in treated BT-474 cells with dihydrotestosterone (DHT) and progesterone. Validation of the top ten differentially androgen-regulated genes and a number of other genes found in enriched signaling pathways was performed by qRT-PCR in BT-474 and other BCa cell lines. In addition, a parallel reaction monitoring targeted proteomic approach was developed to verify selected transcripts at the protein level.

**Results:**

In total 19,450 transcripts were detected, of which 224 were differentially regulated after DHT treatment. The increased expression of two well-known androgen-regulated genes, *KLK2* (p < 0.05) and *KLK3* (p < 0.001), confirmed the successful androgen stimulation in BT-474 cells. The transcription factor, ZBTB16, was the most highly upregulated gene, with ~ 1000-fold change (p < 0.001). Pathway enrichment analysis revealed downregulation of the DNA replication processes (p < 0.05) and upregulation of the androgen signaling and fatty acid metabolism pathways (p < 0.05). Changes related to progesterone treatment showed opposite effects in gene expression than DHT treatment. Similar expression profiles were observed among other BCa cell lines expressing high levels of AR (ZR75.1 and MBA-MB-453). The parallel reaction monitoring targeted proteomic analysis further confirmed that altered protein expression (KLK3, ALOX15B) in the supernatant and cell lysate of DHT-treated BT-474 cells, compared to control cells.

**Discussion:**

Our findings suggest that AR modulates the metabolism of BT-474 cells by affecting the expression of a large number of genes and proteins. Based on further pathway analysis, we suggest that androgen receptor acts as a tumor suppressor in the BT-474 cells.

**Supplementary Information:**

The online version contains supplementary material available at 10.1186/s12014-022-09352-2.

## Background

Despite tremendous efforts in breast cancer (BCa) research, BCa cases are increasing at a rate of 0.3% per year [1]. The expression profile of estrogen receptor (ER), progesterone receptor (PR), androgen receptor (AR) and human epidermal growth factor receptor 2 (HER2) has been used to classify BCa into subgroups, thus facilitating targeted therapies such as the ER^+^BCa most prevalent treatment has been the anti-estrogen drug tamoxifen, whereas for HER2^+^ BCa, the HER2 inhibitor, trastuzumab (Herceptin) is being used. Despite the currently available targeted treatments, drug resistance occurs in many cases after a period, leading to relapse of breast cancer. Cancer cells use various molecular pathways to evade the immune system and drug effectiveness. On the other hand, targeted therapy is restricted to the receptor-positive subtypes since BCa negative in the three main receptors (ER, PR, HER2), also known as triple-negative BCa (TNBC), is treated with chemotherapy.

Existing studies show that the AR affects the ER-expressing BCa through various ways. For instance, AR can bind to estrogen-responsive elements of ER-target genes and modulate their expression [2], competing with ER [3–5]. Thus, the AR/ER ratio sometimes is critical for BCa progression and outcome. For example, when AR is more abundant than ER, AR binds to estrogen responsive elements (EREs) resulting in the decrease of estrogen-mediated proliferation. On the other hand, if ER is expressed at higher levels, ER binds to AREs, inhibiting AR-regulated mechanisms and promoting cell proliferation. In addition, some genes are ER and AR-regulated, such as *MYC*, *GREB1*, *GATA3*, *FOXA1*, *RERG* and *BEX1* [6–8] and their expression (upregulation or downregulation) depends on the receptor status, resulting in promotion or protection against BCa [7–11]. Furthermore, AR affects the ERα and ERβ directly. Specifically, AR binds to the ligand-binding domain (LBD) of the ERα, when androgens are absent and estrogen present. The AR/ERα complex phosphorylates EGFR, promoting DNA synthesis and causing changes in the cytoskeleton [12, 13]. Apart from that, AR binds to the ARE of ERβ, promoting ERβ transcription and the subsequent inhibition of ER signaling and cell proliferation [14–17].

In the present study, we hypothesize that AR and its ligands cause transcriptomic changes in AR^+^ BCa cells. To test this hypothesis, RNAseq analysis was employed in an ER^+^PR^+^HER2^+^ BCa cell line, BT474, with which we could use to identify molecular associations in our study.

We chose RNA seq as a robust, highly sensitive technology, with which we could facilitate new molecular information. The BT474 cell line is a clinically relevant model to investigate the effects of AR (and other candidate molecules that may influence/trigger cancer progression) in BCa, since it was derived from a ductal carcinoma, luminal B type, representative for Stage II cancer of a post-menopausal woman and expresses AR, ER, PR, and HER2 receptor [18–21]. The BT474 cell line is highly expressing AR [19–21]. Once stimulated by androgens (dihydrotestosterone, DHT), BT474 cells produce high levels of KLK3 [22], a well-known androgen-regulated gene, used as a positive control. Our aim was to investigate the AR regulation at the gene expression level of the BCa cell line, BT474. Treatment of BT474 with DHT-mediated expression of several AR-regulated genes and identified molecular pathways that were significantly enriched. Also, BT474 cells were treated with progesterone (PROG) to compare any progesterone-related effects overlapping with androgens [7]. We extended our findings to six other BCa cell lines with various receptor profiles to test the expression of genes that were included in the validation list of BT474. Furthermore, we studied the protein expression of 24 validated mRNAs identified by RNA-seq and investigated their correlation with protein levels using a targeted MS-based approach, parallel reaction monitoring (PRM).

AR expression levels in most BCa cell lines have been determined and this knowledge could be incorporated in existing transcriptomic data to delineate the effect of AR according to the BCa cell types. This information, along with the use of high-throughput techniques (RNAseq and PRM), could facilitate the identification of novel markers for prognosis or personalized therapies.

## Materials and methods

### Cell lines and culture conditions

Seven breast cancer cell lines, BT474, ZR75-1, T47D, MCF7, MDA-MB-453, SkBr3, MDA-MB-468, (ATCC) with different receptor expression profiles were cultured in their respective growth media, listed in Additional file 1: Table S1. The prostate cancer cell line LnCaP was also used as an AR-positive cell line [19], cultured in phenol-free RPMI-1640 medium (Gibco™ ThermoFisher). All media were supplemented with 1% antibiotic–antimycotic and 10% fetal bovine serum (FBS). All cell lines were authenticated with ≥ 80% match (Additional file 3: Table S1-2), at the Centre for Applied Genomics at the hospital for Sick Children (SickKids, Canada).

### Hormonal stimulation of breast cancer cell lines

Cells were cultured in the appropriate growth media (Additional file 1: Table S1) and incubated at 37 °C in an atmosphere of 5% CO_2_ The grown cells were transferred in T-25/T-75 flasks (RNAseq and PRM) and in 24 or 6-well plates (qRT-PCR). Once cells reached the desired confluency, they were subjected to starvation for 24 h using growth media supplemented with 10% charcoal-stripped fetal bovine serum. Next, they were stimulated with dihydrotestosterone (DHT, androgen, 10 nM) or progesterone (progestin, 10 nM) for 24 h and 5 days. The culture supernatants were collected for KLK3 protein quantification using ELISA. Cell lysates were prepared for the mRNA expression study for several genes, using RNA sequencing and real-time qPCR. Stimulation with ethanol was included as a negative control. For each condition, experiments were performed in triplicates.

For the parallel reaction monitoring experiment, starvation was performed using a serum-free growth medium without FBS and insulin. Then, cells were stimulated with ethanol (control, 0.1%) or DHT (10 nM). For the 24 h stimulation, cells were grown at 80% confluency, whereas for the 5-days, cells were stimulated at 60% confluency to avoid cell overgrowth. Cell pellets and supernatants were collected and stored at − 80 °C until further use.

### KLK3 enzyme-linked immunosorbent assay (ELISA)

The samples used were supernatants derived from the hormonally treated cells at 24 h and 5-days, respectively. The KLK3 ELISA was performed as previously described [23].

### RNA extraction and cDNA synthesis

The total RNA was extracted from the cell lines using the RNeasy mini kit (QIAGEN). Total RNA (0.5–1 μg) was reverse-transcribed into first-strand cDNA using the SensiFast cDNA synthesis kit (BIOLINE) for preparing the samples for RNA-seq. The iScript™ cDNA synthesis Kit (BIO-RAD) was used for cDNA synthesis for the validation and inhibition experiments. The RNA extraction and both cDNA synthesis kits were used according to the manufacturer’s instructions. RNA and cDNA concentrations were measured using the NanoDrop spectrophotometer.

### Quantitative RT-PCR

Quantitative real-time RT-PCR was carried out in a 10-μl reaction mixture containing 8 μl SYBR Green Power Up PCR Master mix (Applied Biosystems™), 80–100 ng of cDNA, and 300 nM of each primer according to the manufacturer’s instructions. The conditions for qRT-PCR were as follows: 50 °C for 2 min, 95 °C for 10 min, 45 cycles of 95 °C for 15 s and 60 °C for 1 min.

for melt curve: 95 °C for 15 s, 60 °C for 1 min, 95 °C for 30 s, 60 °C for 15 s. SYBR fluorescence was detected using the ABI 7500 Real-Time PCR and QuantStudio 6 Flex Real-Time PCR Systems (Applied Biosystems). The housekeeping gene Glyceraldehyde-3-Phosphate Dehydrogenase *(GAPDH)* was used to normalize the gene expression data. DHT-treated and control samples were tested in triplicates. The primers of each gene used for validation are listed in Additional file 1: Table S2. The qRT-PCR products were run on 2% agarose gels and visualized by SYBR Safe DNA gel stain.

### RNA sequencing

The BT474 cells were treated with ethanol, DHT or PROG, as described above. The cell pellets were collected after 24 h and washed twice with phosphate-buffered saline (PBS), followed by RNA extraction and clean up (QIAGEN). Samples with 10 μl of 150 ng/μl RNA were sequenced at The Centre for Applied Genomics in the Hospital for Sick Children (Toronto, Canada), using the Illumina Solexa sequencing technology.

#### RNAseq data analysis

The RSeQC package v2.3.7 [24] was used for assessment of the read distribution, positional duplication and to confirm the strandedness of the alignment. Raw trimmed reads were mapped to the human genome hg19 assembly, using Tophat v2.011/Bowtie2 [25]. The reference RPKM (reads per kilobase of transcript per million mapped reads) values were used to estimate gene abundance in each sample. The RPKM values were used to calculate the fold change (FC) between treated and untreated cells. The log_2_-transformed FC values of all genes and distributions were evaluated for DHT vs control (ethanol) and PROG vs control (ethanol) comparisons. The raw gene counts were sample-normalized using DESeq v1.18.0 [26]. Principal component analysis (PCA) was performed to assess the relation among samples.

The statistical analysis of the raw data was performed by the Informatics Facilities at SickKids Hospital (Canada). The EdgeR R package, v.3.8.6 was used for the differential expression analysis. The list of the differentially expressed genes was filtered to only genes whose cpm (counts per million reads) was > 0.4 in at least 2 samples. The significance of the gene expression was calculated based on the Benjamini–Hochberg method (FDR adjusted p-value ≤ 0.05). Further bioinformatics analysis was followed using the Perseus v1.6.0.7, GSEA v.3.0, Cytoscape v3.6.0 (Enrichment Map [27, 28]), and the online tools PANTHER 13.1 [29], Database for Annotation, Visualization and Integrated Discovery (DAVID, 2021 Update) [30], and STRING v11.0 [31] using the default parameters (full STRING network the network edges mean interaction evidence derived from text-mining, experiments, databases, co-expression, neighborhood, gene fusion and co-occurrence interaction score of 0.4).

#### Pathway analysis

The list of differentially expressed genes (DEGs) was used for the pathway analysis. The list was first ranked based on the gene set enrichment analysis (GSEA) score, which was calculated by multiplying the logarithm of the p-value with the direction (sign) of the fold change for each gene [log(p-value) * sign(logFC)] [32].The ranked list was uploaded into the GSEA v.3.0 software and was analyzed using the pathway gene set database (Human_GOBP_AllPathways_no_GO_iea) available by the Bader laboratory (dated May 1, 2018) [27].In order to avoid false-positive gene sets, we used only pathways that contained at least 15 genes and a maximum of 200 genes, 500 permutations and a cutoff of p < 0.1, as suggested by Reimand et al. [32]. The files from GSEAPreranked gene set analysis were uploaded to Cytoscape v3.6.0, using the Enrichment Map plugin for better visualization of the gene set network.

### Inhibition experiments

To further validate the expression of the selected genes, we performed inhibition experiments using two AR-inhibitors.

Enzalutamide (Selleckchem, USA) and hydroxy-flutamide, (Sigma, USA), and one PR-inhibitor (Mifepristone, RU486, kindly provided from Roussel-Uclaf, Romainville, France). BT474 cells were cultured in 6-well plates with the indicated growth media. Once the cells reached 70–80% confluency, they were subjected to growth media supplemented with 10% charcoal stripped FBS for 24 h. Then, they were washed with PBS and incubated with inhibitor compounds for 2 h at a final concentration of 1 μM. The hormonal stimulation was followed by adding the appropriate hormone into the same culture media at a final concentration of 10 nM. After 24 h and 5 days of incubation respectively, cells were collected for mRNA extraction and gene expression analysis with qRT-PCR. The supernatants were collected for KLK3 quantification using ELISA to assess experiment success. Stimulation with 0.1% ethanol was included as a control. All conditions were performed in triplicates.

### Parallel reaction monitoring (PRM) validation experiments

#### Cell culture

Cell pellets and supernatants derived from hormonally treated BT474 cells were collected and processed accordingly for proteomic analysis. Exogenous crude heavy isotopically (^13^C and ^15^N) labeled (heavy) peptides (JPT Peptide Technologies GmbH, Berlin, Germany) were added to the samples during sample processing and used as internal controls to facilitate and ensure the detection of candidate endogenous peptides (light). The PRM method was built with the potential to identify relative quantification of the candidate proteins by calculating the light-to-heavy ratios of the peptide intensities. The process was performed twice (A and B), with triplicated biological samples, at 24 h and 5 days.

#### Sample preparation

The cell pellets were prepared based on a previously described protocol [33]. Supernatant amounts of 15 μg total protein were used for sample assessment, method optimization and sample testing. Once mass spectrometry (MS) confirmed the presence of the tryptic peptides (light), isotopically labeled peptides (heavy) were added at a specific concentration, based on the peptide limit of detection [34]. The heavy (exogenous) and light (endogenous) peptide mixture was then passed through OMIX C18 tips (Agilent Technologies, USA), washed with buffer A (water and 0.1% formic acid) and eluted in 4 μl buffer 65% B (65% acetonitrile, 35% water and 0.1% formic acid). The elution mix was diluted up to 60 μl with buffer A, of which 18 μl w injected into the MS. Samples used for optimization purposes were tested in duplicates. Samples used for protein validation were analyzed in triplicates.

KLK3 expression was assessed in all supernatant samples with ELISA. Despite the KLK3 ELISA results, we considered samples to be evaluable if KLK3 was also detected by MS (limit of quantification 5.7 ng/mL, [34]) and only those samples were used for further data analysis.

#### Peptide selection and method development

Candidate peptides for 23 proteins were selected using the online comprehensive platforms, Selected Reaction Monitoring Atlas (SRMAtlas) [35] and neXtProt [36], which combine peptide information with MS-observed evidence. The peptide selection was based on certain criteria, as previously described [37, 38]. In the end, one to two tryptic peptides per protein that showed a satisfactory chromatographic spectrum in Skyline (v.19.1.0.193) were selected for PRM method development (Additional file 1: Table S5).

The presence of endogenous tryptic peptides was confirmed using 28 crude heavy isotopically (^13^C and ^15^N) labelled peptides (JPT Peptide Technology, Berlin, Germany), which were spiked into light pooled digested samples and injected into the MS. The lyophilized heavy peptides were prepared according to the manufacturer’s guidelines. The peptides were solubilized in 20% ACN and 80% 0.1 M ammonium bicarbonate, at a final concentration of 0.1 nmol/μl, aliquoted and stored at − 20 °C. Small amounts of each heavy peptide were used to create a 1000 fmol/μl (per peptide) heavy peptide pool for optimization purposes. First, 1500 fmol of the heavy peptide pool were added to the 15 μg pooled sample digest in order to determine and verify the retention time (RT) for each peptide. The verified RTs were used for the development of a single multiplexed, scheduled PRM method with a 60-min long gradient. The RT window was set at ± 3 min from verified elution times, with the acquisition of 5–10 points across the peak. The normalized HCD collision energy was set at 27. Due to the two different types of matrices, we developed two PRM methods, one for the proteome samples (cell pellets) and one for the secretome samples (supernatant). In addition, the two PRM methods monitored different proteins/peptides in the proteome and the secretome pools. As a result of the two scheduled PRM methods, we were able to identify nine out of 23 proteins, based on their endogenous presence (light) and the heavy peptide performance within the sample matrix (Additional file 1: Table S6). These nine proteins were present in the final validation list, and their expression levels were studied in the treated vs untreated samples.

### Mass spectrometry

The processed samples were injected with a nano-electrospray ionization source into the Q Exactive HF-X mass spectrometer. The chromatography was performed using the EASY-1000nLC pump (Thermo Fisher Scientific, USA) [39]. The solvent A (buffer A) was water with 0.1% formic acid and the solvent B (buffer B) contained 65% acetonitrile with 0.1% formic acid. The samples were loaded (18 μl) onto a 0.75 µm × 3.3 cm IntegraFrit trap column (New Objective, USA) using buffer A. The peptides were eluted from the trap column using an increasing concentration of buffer B at 300 nl/min onto a resolving 15 cm long analytical column with a PicoTip (8 μm tip) and an inner diameter of 75 μm (New Objective), over a 60 min-long gradient. The columns were packed in-house with Agilent Pursuit C18 media (Aglient, USA) using 5 µm and 3 µm beads for trap and analytical columns, respectively.

For the targeted inclusion list of the PRM method, the MS1 scan range was also 400–1500 m/z at a resolution of 60,000 (at 200 m/z) with AGC target value of 1e6 and maximum IT of 120 ms. The PRM MS/MS filtering had a resolution set to 15,000 (at 200 m/z), AGC target value 2e5, maximum IT time of 120 ms, isolation window of 1.4 m/z with NCE set to 27, inclusion mass accuracy of 10 or 5 ppm, and acquisition duration of 6 min for each peptide. The 60-min gradient for the PRM consisted of 0–2 min 1–5% buffer B, 2–49 min 5–35% buffer B, 49–52 min 35–65% buffer B, 52–53 min 65–100% buffer B at a 300 nL/min flow rate. At 53 min, the flow rate increased to 450 nL/min within 10 s and remained at this flow rate for seven additional minutes until the end of the gradient.

### MS bioinformatic analysis and statistics

The Xcalibur software v.4.3.73.11 (Thermo Fisher Scientific, USA) was used to generate raw files. The raw files were uploaded to Proteome Discover (PD) v1.4 and searched with the SwissProt human search engine for determination and identification of proteins. The false detection rate (FDR) for the PD searches was set to a high confidence level (1% FDR). Skyline software (v. 19.1.0.193) was used simultaneously for the identification and visualization of transitions and determine the presence and co-elution of heavy and endogenous peptides at the same retention time (RT). We examined all peptides manually for the accurate integration of their peaks and the quantification of the area-under the curve (AUC) was used for the calculation of the light to heavy peptide ratios (AUC_light_/AUC_heavy_).

Statistical analysis was carried out using the GraphPad Prism v.8.0 software (GraphPad Software, San Diego, CA). The statistical significance was determined using the student’s t-test for each protein, between the two conditions (treated vs untreated samples), without correction for multiple comparisons and alpha = 0.05. The protein expression was analyzed individually without assuming a consistent SD. P-values less than 0.05 were considered statistically significant.

## Results

### Investigation of androgen-regulated changes in the BT474 transcriptome

**Fig. 1:**
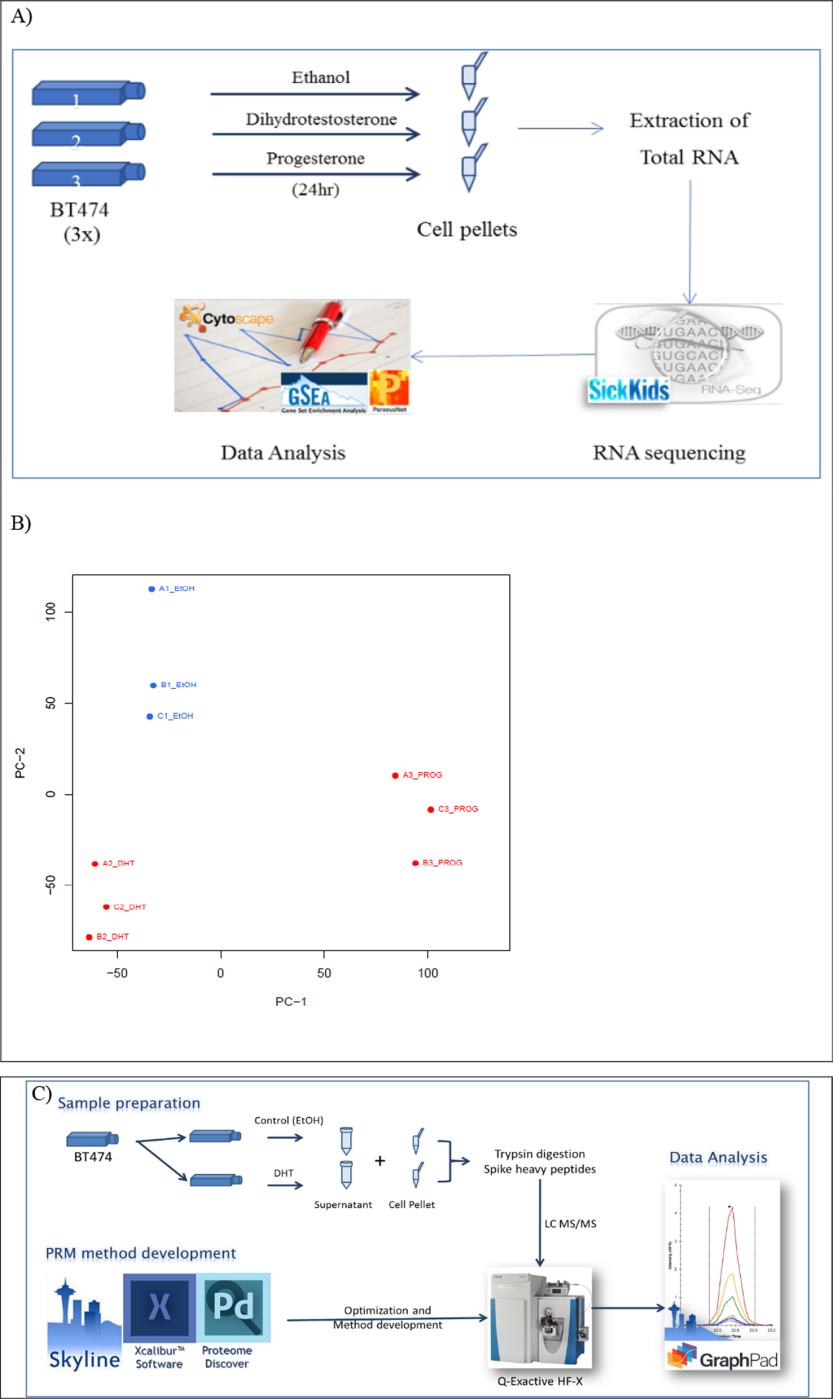
Project pipeline. **A** Workflow of the transcriptomic analysis of hormonally treated BT474 cells. Cells were cultured and treated with hormones or ethanol (used as control) for 24 h. Cell pellets were collected and subjected to total RNA isolation. GSEA and Cytoscape software was used for the functional and pathway analysis of the differentially expressed genes. DHT dihydrotestosterone (10 nM), PROG progesterone (10 nM). **B** Principal Component Analysis (PCA) of BT474 RNA sequencing in three treatment conditions (Ethanol, DHT, and PROG). Each condition was performed in triplicates and groups clearly cluster. DHT dihydrotestosterone (10 nM), PROG progesterone (10 nM). **C** Experimental workflow of targeted PRM proteomics and data analysis for the validation of select androgen-regulated proteins in the BT474 cell line. For details see under “methods”

Our aim was to search for transcriptomic changes in the BT474 cell line upon androgen stimulation. First, we investigated whether BT474 cells responded to the treatment of the two hormones, DHT (10 nM) and progesterone (10 nM), by examining mRNA levels of the positive control, the well-known androgen-regulated gene, *KLK3*. Only DHT induced the expression of *KLK3*, whose mRNA levels were upregulated after 24 h stimulation (Additional file 2: Fig. S1A). We then continued with RNAseq analysis (Fig. 1A).

Samples collected after the 24 h treatment were subjected to total RNA extraction and RNA sequencing was conducted using the HiSeq Illumina Technology platform (Fig. 1A). The starting reads were ~ 30 million, which were processed (trimming of adaptors and low quality) and aligned to the genome. The libraries constructed contained minimal amounts of mtRNA, 92% of the reads mapped to exons, and the final number of reads for analysis was 21–24 million, a depth suitable for differential expression analysis. In addition, an empirical permutation-based procedure PCA (Principal Component Analysis) was performed to assess a relation among all samples and identify informative principal components. In this analysis, the informative components of the PCA are PC1 and PC2 (Fig. 1B). The first principal component (PC1) corresponds to the most significant factor that differentiates the samples, representing the cells' response to individual treatment the samples grouped well by condition (ethanol, DHT, PROG). The PC2 describes the second critical factor, corresponding to the hormonal (DHT and PROG) versus control (ethanol) treatments. This observation seems reasonable since the hormonal treatments are expected to stimulate pathways that would not be activated by ethanol alone. In addition, the inter-group variability was small since the three replicates of each condition grouped close together. This distinct grouping provided a better comparison of the transcriptomes between the three groups and enabled us to continue with further analysis (Fig. 1B). As a result, the data analysis can be performed with confidence that any difference observed between treated and untreated cells is related to the hormone effect on the cells and is not a random finding.

**Fig. 2:**
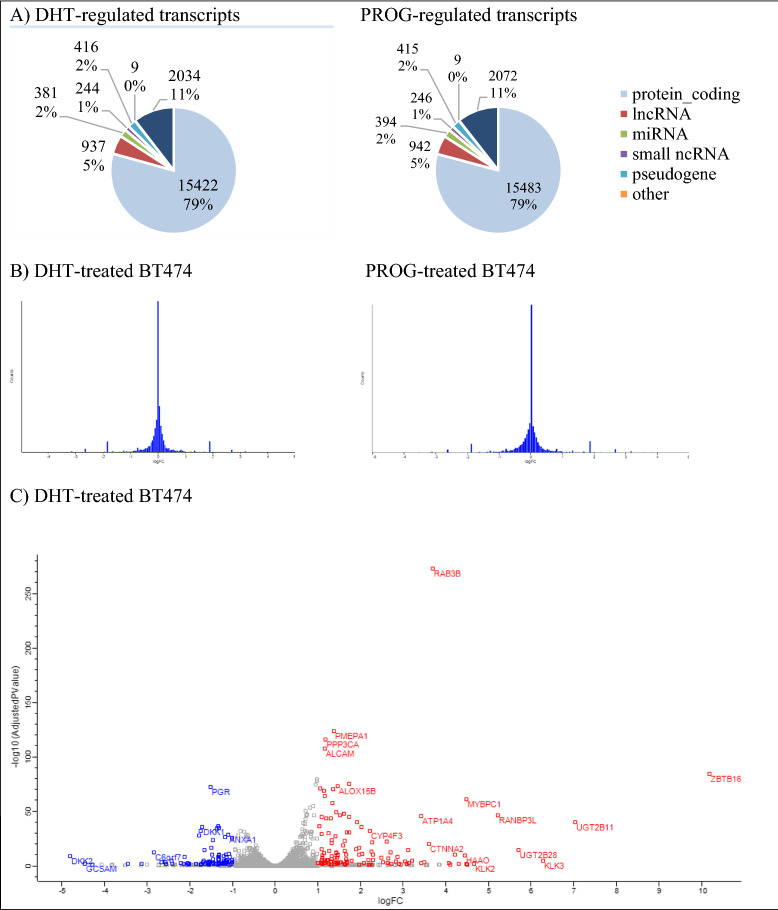

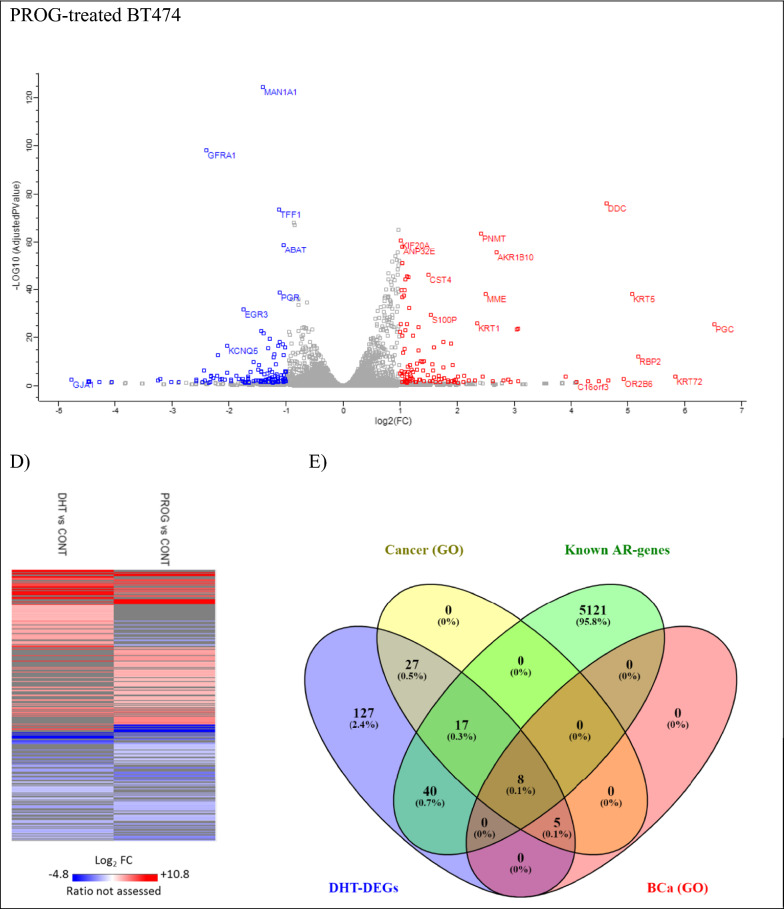

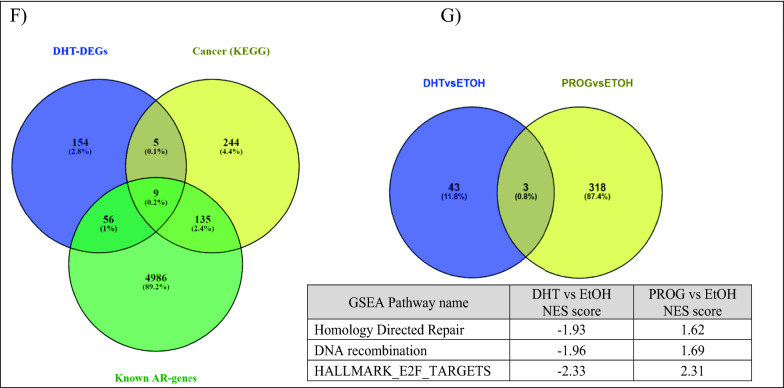
Hormonally regulated changes in the BT474 transcriptome. **A** Pie charts illustrating the different transcript types that are hormonally regulated in BT474 cells. In both conditions, DHT- and PROG-treated cells, 79% of the transcripts are protein-coding, 5% long non-coding RNAs (lncRNA), 2% microRNAs (miRNA), 2% small non-coding RNA (ncRNA), 2% pseudogenes, and the remaining 11% belong to other categories and gene ID duplications. *snoRNA, snRNA, scaRNA, scRNA, miscRNA. **Polymorphic_pseudogene, processed_pseudogene, transcribed_processed_pseudogene, transcribed_unitary_pseudogene, transcribed_unprocessed_ pseudogene, unitary_pseudogene, unprocessed_pseudogene. ***TEC (To be experimentally confirmed), ribosome The transcript categorization was performed on Ensembl website, using the bioinformatics tool, Biomart. **B** Histograms demonstrating the FC (fold change) distribution of DEGs in the transcript list, **C** Volcano plots illustrating the most significant differentially expressed genes. Red color shows the upregulated genes and blue color shows the downregulated genes. **D** Heatmap illustrating the differential expression profiles in DHT-treated and PROG-treated cells, respectively, compared to controls. Red color represents the upregulated genes, whereas the blue color represents the downregulated genes. Grey color corresponds to the genes that were not expressed, or the ratio could not be assessed in this experiment. All graphs were constructed in Perseus software using the log_2_FC values derived from the expression comparison between the hormone-treated and control cells. **E**, **F** Venn diagram representing the number and overlapping of DEGs in DHT-treated BT474 cells with known AR-regulated and cancer/BCa-related genes. The identification of the cancer/BCa related enriched genes were identified with the Gene Ontology annotation (**E**) and the KEGG analysis (**F**) of DAVID (2021 Update) online tool. **G** Comparison of gene set numbers identified in GSEA analysis of the RNA-seq experiment in DHT and PROG-treated BT474 cells. For the GSEA (v.3.0) analysis, only gene sets that contained 15–200 genes were used (q-value < 0.1). DHT dihydrotestosterone (10 nM), PROG progesterone (10 nM) *NES: normalized enrichment score

We identified 19,443 differentially expressed genes (DEGs) between the DHT-treated BT474 and control cells, whereas in the PROG-treated BT474 cells, 19,561 DEGs were identified. Of those genes, 15,422 and 15,483 genes respectively, were protein-coding genes (Ensembl, Biomart analysis [40], Fig. 2A), resulting in a significant portion (~ 77%) of the whole genome [41].

The histograms illustrating the normal distribution of differentially expressed transcripts (FDR ≤ 0.05), a heat map and volcano plots were generated (Fig. 2B–D) using Perseus software. The heatmap showed that the gene expression profile of the DHT-treated cells differs from the one derived from PROG-treated cells. Many of the genes expressed in DHT-treated cells are not differentially expressed in PROG-treated cells (grey color) (Fig. 2D) and others show the opposite direction of expression (up vs downregulation), and vice versa. This finding suggests that cells respond to the two hormones differently.

We only considered genes that showed more than two (≥ 2) fold-change in RNA abundance for further analysis and higher confidence. In DHT-stimulated BT474 cells, we identified 224 significantly differentially expressed genes, of which 132 were up-regulated and 92 were down-regulated (Table 1). Nine genes were non-detectable before treatment and were detected only after the DHT stimulation. In addition, six genes were silenced after DHT treatment, since no transcripts were detected post-treatment (raw count was 0 in all replicates). A brief analysis of these 224 genes showed that 195 of them were protein-coding, and 29 were non-coding transcripts (Table 1). Treatment with progesterone resulted in 243 differentially expressed genes, of which 122 were up-regulated and 121 were down-regulated (Table 1). Similar to the DHT treatment, nine genes were expressed only after treatment, whereas 10 genes were silenced. Among the 243 genes, 214 were protein-coding, and 29 were non-coding (Table 1).

**Table 1:** Summary table of DEGs in hormonally treated BT474 cells with RNA sequencing

	DHT vs EtOH	Progesterone vs EtOH
Genes detected	19,443 (15,422)^a^	19,561 (15,483)^a^
Genes significant (EdgeR)	224 (195)^a^	243 (214)^a^
Common significant genes	45	
Upregulated	132 (9)^b^	122 (9)^b^
Downregulated	92 (6)^b^	121 (10)^b^

^a^Protein-coding transcripts

^b^Transcripts identified in terms of presence vs absence, and vice versa (see text)

Comparison of the DEGs between the DHT and progesterone responses showed that only 45 genes (10.7%) were common. Among the 45 DEGs, 18 were upregulated, 21 were downregulated, and 6 were DHT-upregulated and PROG-downregulated (Additional file 2: Fig. S2D). The complete list of the significant DEGs as a result of the hormonal treatment of BT474 cells is shown in Additional file 1: Table S4.

### Gene ontology and pathway analysis reveals cancer-related pathways gene expression

The gene ontology (GO) analysis of the differentially expressed transcripts was performed using the publicly available classification system PANTHER [29]. Results showed similar patterns of GO analysis between the two conditions for the molecular function, biological process, and cellular component of DEGs (Additional file 2: Fig. S2). Around 40% of the DEGs participate in “catalytic activities” (Additional file 2: Fig. S2A), consistent with the increased metabolic processes (Additional file 2: Fig. S2B). Biological processes that show higher enrichment in the DHT-treated cells include developmental processes and biological regulation, which are also found to be significant by other androgen-related reports [42]. In the cellular component GO analysis, the DHT enhanced the expression of more genes that are associated with the membrane component, which is also consistent with the increased activation of membrane transporter proteins.

Additional signaling analysis was performed using DAVID (2021 Update) functional annotation. Of the 224 significantly DHT-regulated DEGs, 57 genes were significantly enriched in the GAD_DISEASE_CLASS category, under the term “cancer” (Additional file 1: Table S7). Interestingly, 13 of the 57 genes (*ABCC11*, *BCL2*, *CXCL12*, *UGT1A6*, *UGT2B11*, *UGT2B15*, *ADRB2*, *ADH1C*, *DRD2*, *IGF1*, *KLK3*, *PNMT*, and *PGR*) were associated with BCa. Further analysis in DAVID with the Kyoto Encyclopedia of Genes and Genomes (KEGG) revealed twelve KEGG pathways that were found to be significantly enriched (Benjamini–Hochberg FDR ≤ 0.05), whereas under PROG conditions no significant enrichment pathway was identified (Additional file 1: Table S8). In DHT-enriched pathways, the cancer-related pathways contained the highest number of genes (14 genes, 6.5%) compared to the other significant pathways. The comparison of the above- mentioned genes with a list of 5,186 known AR-regulated genes (Additional file 3: Table S3), showed that nine (*ZBTB16, KLK3, IGF1, NKX3-1, CTNNA2, CXCL12, WNT6, WNT10B, and BCL2*) out of the 14 cancer-related genes are known to be AR-regulated, whereas the rest five (*ADCY2, LAMA3, LAMC2, RARB*, and *PTGER3*) are identified here as newly AR-regulated candidates.

Furthermore, a total of 159 genes were first identified as AR-regulated genes (cancer and non-cancer related) in this study (Fig. 2E). In the top 20 upregulated DEGs, eight genes (*CYP4F8*, *MIR548D1*, *HAAO*, *OR2B6*, *ESPNP*, *CASQ1*, *C11orf91*, and *TDRG1*) were identified as newly AR-regulated genes (Additional file 1: Table S4). This finding suggests that AR acts differently in various BCa cells and underlines the variation of the hormonal effect in various cancerous cells. Here we show that AR can target unknown cancer-related genes and affect their expression, which may be associated indirectly with molecular pathways related to cancer or anti-cancer phenotypes. Thus, under a more global point of view of the AR function in this BCa cell line, we further analyzed our data with the GSEA method.

#### Pathway analysis of the hormonal regulated DEGs

**Fig. 3:**
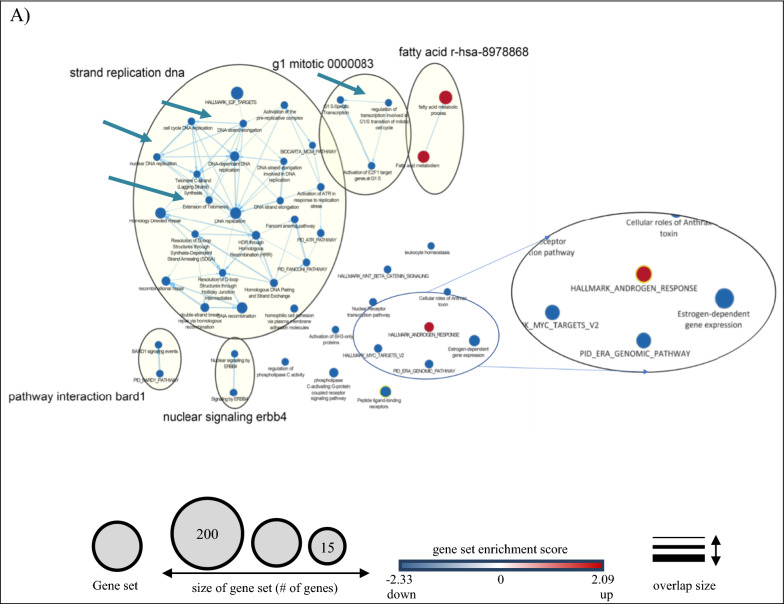

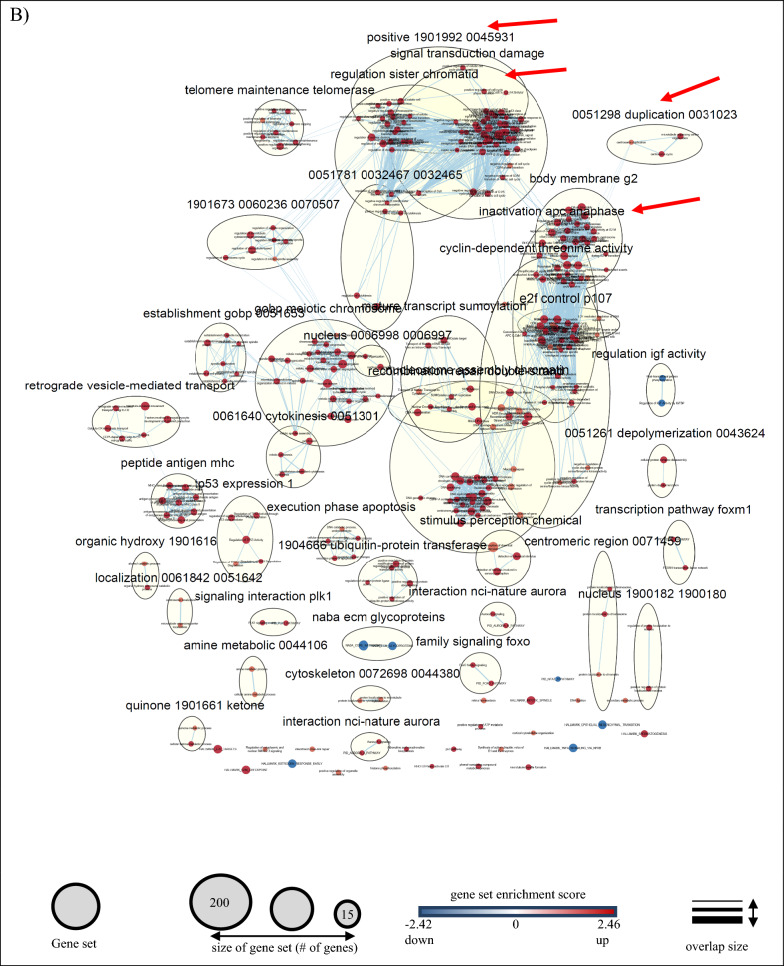
Pathway analysis of hormone-regulated genes identified by RNA sequencing of BT474 cells. Enrichment map of the DHT- (**A**) and PROG- (**B**) regulated genes. The analysis was performed using the Cytoscape software (GSEA 3.0, Enrichment Map plugin, q-value < 0.1). Blue color nodes represent downregulated gene sets, whereas red color nodes show the upregulated gene sets. The node size corresponds to the number of genes included within each gene set. For details see under Section “methods”

In order to reveal the biological significance of our transcriptomic results, we performed a GSEA analysis to identify gene sets and enrichment signaling pathways associated with BCa. We identified 46 enrichment pathways, of which only three gene sets were upregulated (1 of androgen-related pathway and two gene sets that belong to the fatty acid metabolism (FAM), and 43 downregulated (Additional file 3: Table S6). An enrichment map (q < 0.1) was generated to visualize the enrichment network pathways (Fig. 3A). Two or more gene sets that showed a good relationship were clustered together and annotated under the same group.

On the other hand, GSEA analysis of the PROG-regulated data set resulted in 321 enriched gene sets (Fig. 3B), 313 of which were upregulated and eight downregulated. Comparison of the gene sets identified in DHT and PROG-treated cells (Fig. 2G) revealed only three common gene sets. However, these three pathways display opposite enrichment score in DHT (negative) and the PROG (positive) conditions (Fig. 2G). In addition, using the AutoAnnotate Cytoscape application, we grouped gene sets into major biological themes based on the frequency within the pathways. We observed that clusters related to DNA replication and proliferation consist of downregulated pathways under DHT-treatment (blue arrow in Fig. 3A), whereas in PROG-treatment they are upregulated (red arrow in Fig. 3B). This observation confirms the role of DHT as an anti-proliferative hormone in ER^+^PR^+^ BT474 cells in contrast with progesterone, which promotes cell proliferation and progression in BCa cells [43].

We further analyzed the gene sets identified in DHT-treated samples by investigating the leading-edge gene subset of the top 20 gene sets, according to the normalized enrichment score (NES). We found that 20 genes included in the leading edge of those gene sets (Additional file 1: Table S9) were significantly expressed (significant DEGs list). Among these, *AZGP1* was included in the leading edge of the “Hallmark androgen response” gene set and is a known AR-target gene [44, 45]. However, this gene is known to stimulate lipid degradation (UniProt [46]), suggesting that *AZGP1* may act as a mediator between AR and the FAM, since other seven significant genes are involved in this pathway (*CYP4F3*, *CYP4F12*, *CYP2A6*, *ALOX15B*, *CROT*, *ACSM1*, and *HPGD*). Other genes that are not included in the leading edge of the top 20 gene sets, but are also FAM-related, are listed on Additional file 1: Table S10. Moreover, *CROT* and *HPGD* are known androgen-regulated genes in PCa [47], but were not included on the leading edge of the “Hallmark androgen response” gene set. Functional information for those genes was obtained from UniProt [46]. Interestingly, *AZGP1* (FC: 4.84) and the rest of the genes were found to be positively regulated by DHT (Additional file 1: Table S4).

### In vitro validation of candidate genes with quantitative real-time PCR

To validate our RNAseq findings we used quantitative real-time PCR. A list of candidate genes for validation (Table 2 and Additional file 1: Table S12) was created based on two criteria. The first criterion includes the top 10 protein-coding upregulated genes (#1–10) and two top downregulated genes (#20–21) of significantly DHT-regulated transcripts (Table 2). The second criterion included genes that belong to selective enrichment pathways (#11–19). Two additional genes, *S100P* (#22) and *NDC80* (#23), were tested as non-DHT-regulated (controls), since they showed a significant increase (> 2-FC, p-value < 0.05) only in PROG-treated cells. For example, *S100P* is a known PROG-regulated gene [48]. The expression of *AR* (#24) and *ESR1* (estrogen receptor 1) (#25) was also tested.

**Table 2:**
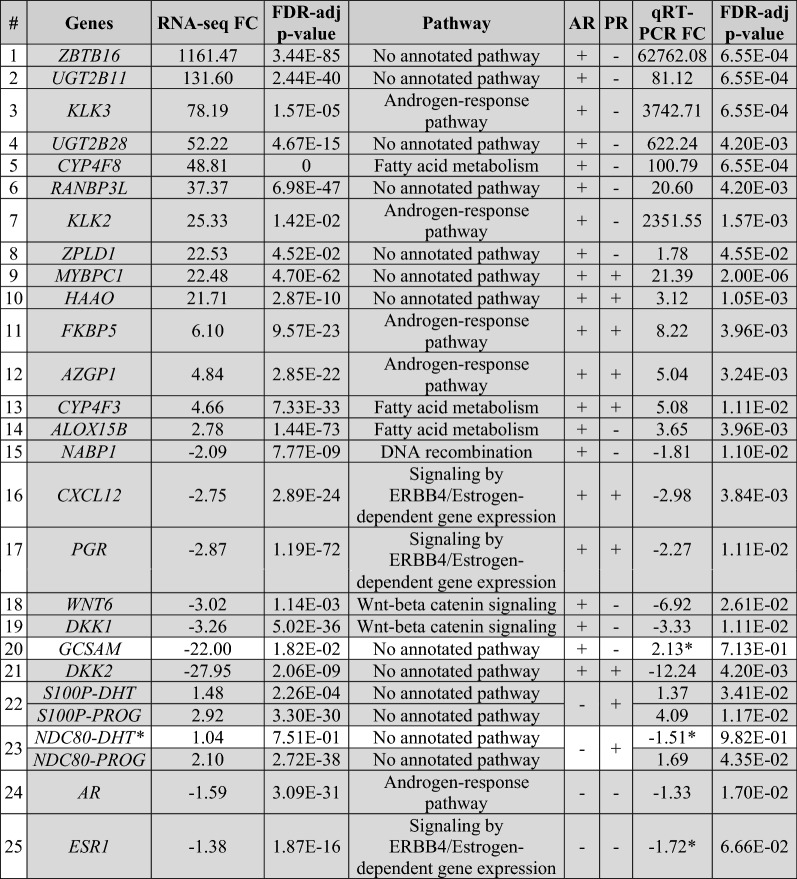
List of candidate genes for validation of RNAseq

Rows highlighted in grey indicate the validated genes by qRT-PCR

*FC* fold change

The expression of almost all the candidate genes was confirmed by qRT-PCR. All genes showed the same direction of expression in both RNAseq and qRT-PCR (Additional file 2: Fig. S3A), except *GSCAM*. As expected, the four additional genes (*S100P, NDC80, AR, and ESR1*) did not show significant change (> ± 2 FC, p-value > 0.05) in DHT-treated cells. In both PROG-regulated genes, *S100P* and *NDC80*, their expression was also validated with qRT-PCR as shown in Additional file 2: Fig. S3B. Depiction of representative validated genes that their FC expression could be shown qualitatively is shown in Additional file 2: Fig. S3C.

Inhibition of AR suppressed five genes, (*ZBTB16*, *KLK3, UGT2B11*, *UGT2B28* and *MYBPC1)* (Additional file 2: Fig. S4C), which are included in the top 10 highly upregulated genes in DHT-treated BT474 cells (Table 2). At the same time, two representative AR-downregulated genes, (*CXCL12* and *PGR*), showed significantly increased expression levels after the inhibition of AR (Additional file 2: Fig. S4C).

### BCa cell lines with high AR expression levels share similar expression profiles

Our results showed that AR expression levels of MDA-MB-453 cells are the highest [49], followed by BT474, ZR75.1, whereas SkBr3 cells expressed the lowest AR mRNA levels. This is also in agreement with the RNA sequencing data available from CCLE (Cancer Cell Line Encyclopedia, https://sites.broadinstitute.org/ccle/) (Additional file 1: Table S11 and Additional file 2: Fig. S5A). Analysis of the expression of the validated genes revealed that BT474, ZR75.1 and MDA-MB-453 cell lines share common expression changes in many genes (Additional file 2: Fig. S5E). Interestingly, these three cell lines have the highest levels of AR compared to the other cell lines. Furthermore, we classified the validated genes based on the GSEA pathway analysis, to identify any pathways that are enriched in the above-mentioned cell lines (Additional file 2: Fig. S6). Expression of most genes (*ZBTB16*, *UGT2B11*, *UGT2B28*, *RANBP3L*, *MYBPC1*, and *HAAO*) were not included in any annotated pathway. These genes along with the FAM-related genes were found to be increased in the three AR-highly expressed cell lines. Depiction of representative validated genes and their FC expression could be found in Additional file 2: Fig. S6B.

### PRM verifies the AR-regulated expression of the top candidate genes

A general representation of the PRM experimental workflow is depicted in Fig. 1C. KLK3 expression was tested in treated and untreated BT474 cells, at the protein level, as indicator (positive control) of the successful stimulation. As expected, KLK3 protein levels increased after 5-days in both independent experiments, A and B (Additional file 2: Fig. S1B). We investigated the protein expression of 23 genes in BT474 of these 21 were qRT-PCR-validated genes (identified in RNA sequencing) two genes were used as additional controls including *PMEPA1* (known AR-regulated gene, second positive control at 24 h) [50, 51] and *NDC80* (cell cycle and mitosis pathway, negative control at 24 h) [52] (Table 2, Additional file 1: Table S12). Nine out of 23 (39%) proteins were verified (Additional file 2: Fig. S7A). Four proteins (KLK3, ALOX15B, AZGP1, and S100P) showed increased expression after DHT treatment, whereas five proteins (PGR, NABP1, NDC80, CXCL12, and DKK1) showed a decrease.

It is noteworthy that in some cases, the L/H ratio could not be calculated due to the absence of endogenous (light) peptides. (Additional file 2: Fig. S7B, C.). Some of those proteins are grouped under the same biological pathways and behaved as expected. For example, KLK3, AZGP1, and IQGAP2 belong to the upregulated androgen-responsive pathway, and PGR, and CXCL12 belong to the downregulated estrogen-dependent gene expression pathway (GSEA, Enrichment map). In addition, DKK1, member of WNT-beta catenin signaling pathway, and ALOX15B, a member of fatty acid metabolism, were found to be downregulated and upregulated, respectively, consistent with the negative/positive enrichment of the respective pathways (GSEA analysis). Similarly, genes associated with cell cycle were found to be downregulated for example, NABP1 belongs to the DNA recombination/repair process (negatively enriched, GSEA analysis of RNAseq) and NDC80 (member of kinetochore complex) is required for the spindle checkpoint during mitosis [52, 53].

### Interaction studies

**Fig. 4:**
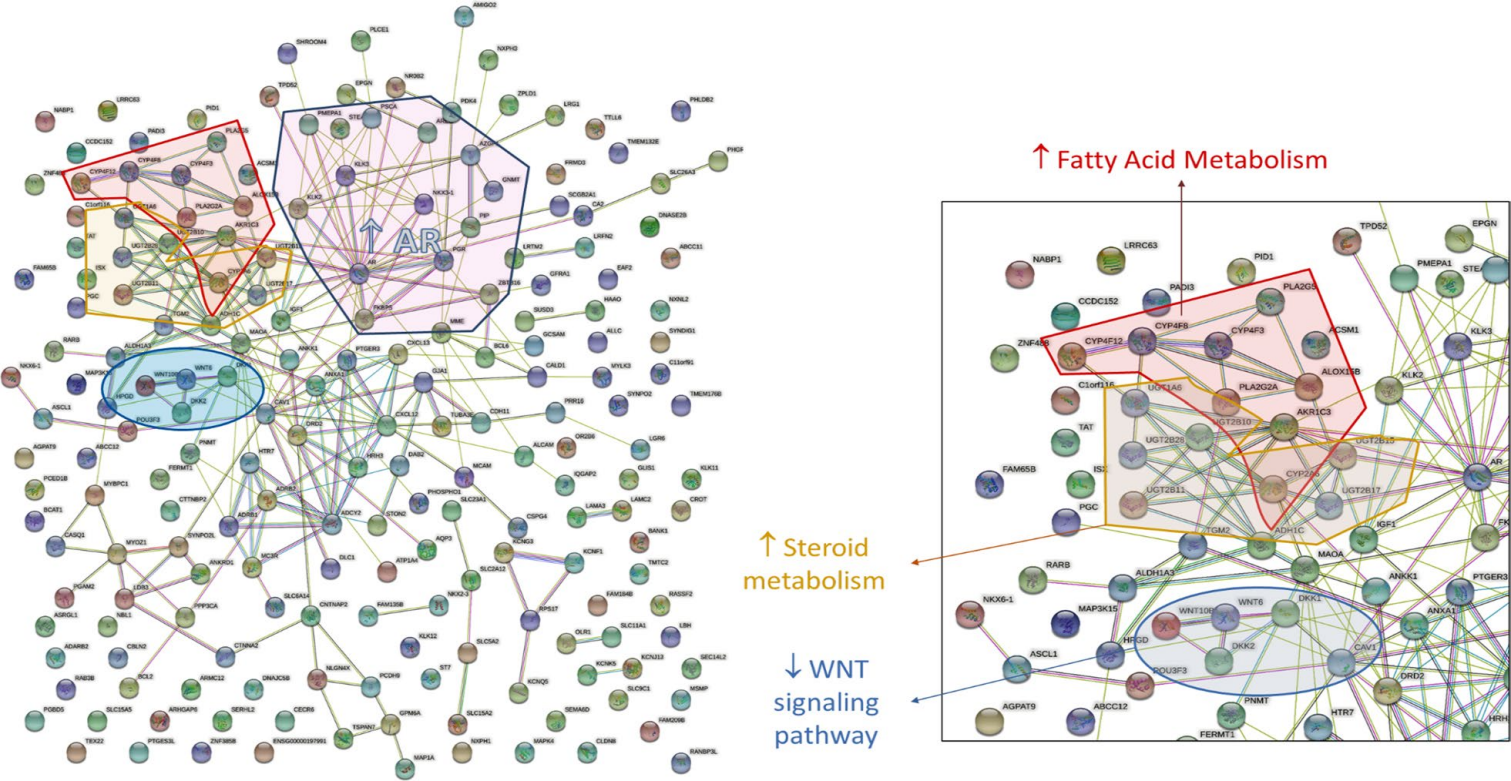
Schematic representation of the significant DEGs of DHT-regulated BT474 cells, using the online tool, STRING. The genes associated with either fatty acid metabolism or the WNT-pathway, cluster clearly together. Lines with different colors represent the source of data that indicate the protein–protein interaction light blue: from curated databases, purple: experimentally determined, green: gene neighborhood, red: gene fusions, dark blue: gene co-occurrence, yellow: text mining, black: co-expression, and grey: protein homology

The STRING analysis confirmed connection of co-expressed genes, mentioned above (Fig. 4). Some examples are AR, KLK3, and AZGP1, which belong to the androgen-response pathway and PGR, and CXCL12, which are known members of estrogen-dependent gene expression and interact directly with ESR1. It is worth mentioning that the gene group, AR-KLK3-AZGP1-(STRING network, Fig. 4), was mentioned in a patent application as biomarkers for PCa diagnosis in 2015 [54], and were also detected in proteomic studies of seminal plasma [45], suggesting that there is a strong androgen-dependent association among these genes. Moreover, high protein levels of AZGP1 were also associated with less aggressive phenotype in ER^+^ BCa and delayed recurrence of PCa [55].

### Correlation of the transcriptomic and proteomic changes of DHT-treated BT474 cells

**Fig. 5:**
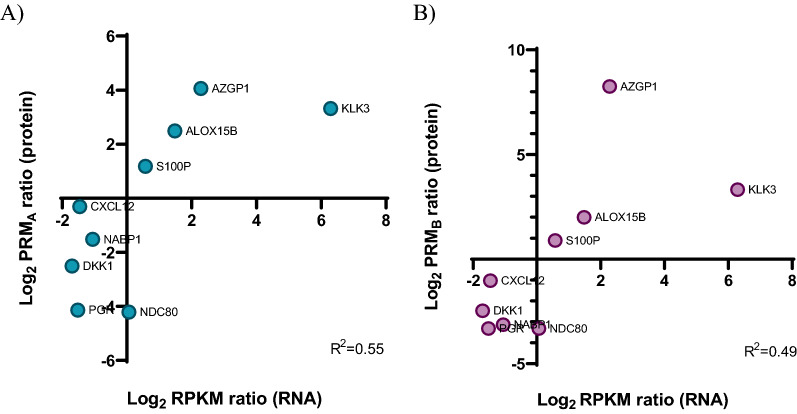
Pearson correlation analysis between the transcript and protein levels of DHT-regulated genes in BT474 cells. The X axis shows the transcriptomic (log_2_ RPKM ratio) and the Y axis the proteomic (log_2_ PRM ratio) levels of DHT-treated BT474 cells. The graphs **A** and **B** show the comparison of the RNA sequencing results with the two PRM experiments, respectively. Pearson correlation was calculated using the GraphPad software

Next, we wanted to examine if protein levels correlated with mRNA expression of the respective genes. We performed a Pearson correlation analysis between the log_2_ fold change values of the 17-verified proteins by PRM experiments (A and B) and the log_2_ RPKM values derived from RNA sequencing. In both comparisons (RPM_A/B_ vs RNAseq), the correlation coefficient (r) showed a moderate to strong positive linear relationship (r_A_ = 0.73 and r_B_ = 0.69 respectively, Additional file 1: Table S13) [56], suggesting that for these 17 genes, mRNA expression changes were accompanied with similar protein levels changes (Fig. 5A and B).

## Discussion

Increasing technological advances helped to improve our knowledge in BCa-related molecular characteristics [57]. Although cost prohibitive as a routine molecular diagnostic for the clinic, RNA sequencing has proved a powerful tool. Therefore, BCa research with RNA-seq in tissue samples or cell lines can help focus on molecular mechanisms of cancer progression.

In our study, we used RNA sequencing to identify androgen-regulated genes in the BCa cell line, BT474, and investigated transcriptomic changes caused by the androgen, DHT. The BT474 cell line is an ER^+^PR^+^HER2^+^AR^+^ BCa cell line with high response to androgen treatment [18, 22] and for this reason, we chose it as a model for the current experiments. Of the 19,443 candidate DHT-regulated DEGs, 15,422 (79.3%) were protein-coding transcripts. As observed, AR and its ligand, DHT, affect the expression of known and unknown androgen-regulated genes associated with cancer (Fig. 2E, F, Additional file 3: Table S4-5), many of which are also BCa-related genes. At the same time, we investigated the effect of progesterone to exclude the possibility that any observed changes can be caused non-specifically by either of the two hormones. While comparing the results of the two hormonal treatments, we found that a few identical genes exhibited similar expression (9.3%, Fig. 2D, Additional file 2: Fig. S2D), whereas the molecular pathways were enriched in opposing directions (Fig. 2G).

The results from GSEA analysis showed that only three gene sets were positively enriched in DHT-treated cells. The first one was the androgen-related pathway and the other two belonged to fatty acid metabolism (Fig. 3A). The enrichment of the first pathway was somehow expected since the observations were seen upon stimulation with the androgen DHT. The second molecular pathway, the fatty acid metabolism, was an interesting finding. The fatty acid metabolism (FAM) is an essential component for energy production in cells. Dysregulation of FAM has been associated with a malignant phenotype in different cancer types, such as PCa [42] and BCa [58]. Monaco M.E et al. [58] attempted to gather information regarding FAM pathways and classify them based on the intrinsic molecular BCa subtypes. The authors report that the luminal-type BCas maintain a balance between lipid synthesis and oxidation, whereas the more aggressive basal-like BCa, TNBC, overexpress genes that are related to the utilization of exogenous fatty acids. Similarly, our data show overexpression of genes associated with fatty acid synthesis (*ACACA, FASN)* and activation (*ACSL3*), and downregulation of *SLC6A14*, which is involved in glutamine uptake. Another study reports the upregulation of *FASN* expression upon DHT stimulation in AR^+^ER^+^PR^+^T47D cells, which are accompanied by the formation of lipid droplets and decreased cell growth [59]. In addition, the known AR-upregulated genes, *UGT2B11* and *UGT2B28*, were recently reported to cross-talk between androgen and lipid signaling [60, 61]. Thus, DHT could cause lipid accumulation, which leads to cell differentiation by arresting the proliferation of cancer cells through gene regulation [59].

**Fig. 6:**
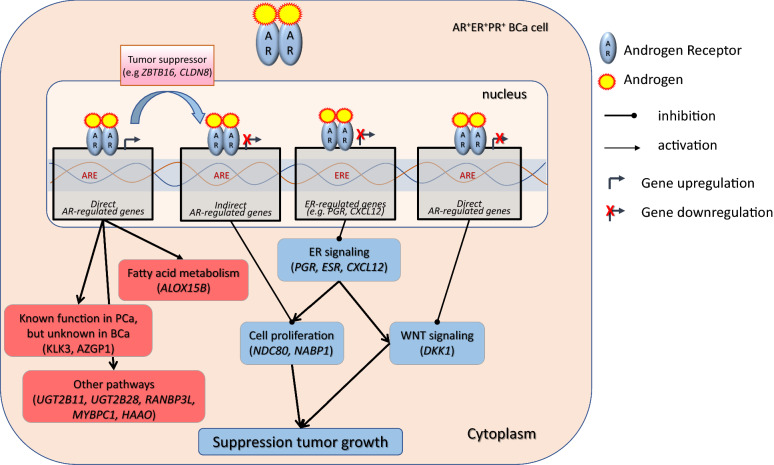
Proposed action of AR in the AR^+^ER^+^BCa cell line, BT474. Treatment of BT474 cells with the androgen DHT caused upregulation of known and unknown A-regulated genes, including genes related to fatty acid metabolism and tumor-suppressor genes, such as *ZBTB16* and *CLDN8*. At the same time, AR suppressed directly or indirectly the expression of genes related to various pathways, such as WNT signaling, and ER-responsive pathways, resulting in decreased cell proliferation and tumor growth. Boxes in red and blue color represent upregulated and downregulated pathways, respectively

The arrest of cell proliferation was observed through the GSEA analysis (Fig. 3A), in which most of the downregulated pathways were associated with DNA replication, mitosis, and other proliferation-related pathways. Two representative genes, *NDC80* and *NABP1*, were found to be downregulated by DHT. Previous studies showed that NDC80, a vital member of the kinetochore assembly complex during mitosis, was upregulated in benign breast tumors [53] and was related to the malignant features of BCa [62]. On the other hand, the role of *NABP1,* a gene associated with DNA replication (GSEA analysis), in BCa is still unclear. However, by reducing the expression of all those genes, AR may suppress cell proliferation (Fig. 6), suggesting a protective role in BCa progression in BT474 cells, and perhaps in other AR^+^ER^+^ BCas, that needs to be further investigated.

Furthermore, the upregulation of two tumor suppressing genes, *CLDN8* (FC: 3.34) and *ZBTB16* (FC: 1161.47) is in concordance with the above finding. The first gene, *CLDN8*, is associated with a favorable prognosis in BCa when it is co-expressed with AR, suggesting that *CLDN8* acts as a tumor-suppressor gene [63]. In the same way, the top DHT-upregulated gene, *ZBTB16*, is a known AR-regulated gene mainly for its expression in PCa. In BCa, *ZBTB16* was also found to be expressed, but little is known regarding its function [7]. This gene is a tumor-suppressor with anti-proliferative activity in PCa cells [64]. Androgen receptor causes *ZBTB16* expression in PCa cells and in turn ZBTB16 regulates AR activity by acting as a negative feedback regulator, controlling AR-dependent cell proliferation [65–67]. Considering this, it is possible that AR upregulates *ZBTB16* expression to suppress the expression of downstream genes that promote the cancerous behavior of these cells.

In addition, we validated the downregulation of genes related with the Wnt-pathway (*DKK1* [GSEA], *WNT6* [GSEA], *WNT10B* [GSEA], and *CAV1* [68]) and estrogen-pathway (*PGR* and *CXCL12*), which are usually increased in ER^+^ BCa [69] (Fig. 3A and Additional file 2: Fig. S3A). Furthermore, it is worth mentioning that *DKK1* (UniProt [46]) and *BCL2* genes [70] show anti-apoptotic activity. Thus, their DHT-induced downregulation may activate the apoptotic process in BCa cells. Last but not least, *MYBPC1,* which is a known AR-regulated gene in PCa [47], was found to be one of the top 10 upregulated genes. Interestingly, it is suggested to be a favorable prognostic marker in BCa based on the Human Protein Atlas Pathology [71].

Another important finding was identifying a common gene expression profile in three BCa cell lines, BT474, ZR75.1 and MDA-MB-453, after DHT treatment (Additional file 2: Fig. S5). The first two are AR^+^ER^+^PR^+^ BCa cell lines, whereas the third is an AR^+^TNBC. In agreement with the literature [19, 72, 73], we found that all the three cell lines express AR at higher levels in comparison to the other BCa cell lines (Additional file 1: Table S1 and Additional file 2: Fig. S5A).

Verification of the candidate genes using PRM further supports our data from RNAseq. The expression of nine out of 23 genes (39%) was verified with a positive correlation between their mRNA and protein expression levels in both PRM experiments (Fig. 5). However, this percentage is low, but it was expected considering the challenges of low correspondence between mRNA and protein expression levels due to biological (e.g., molecular differences between mRNA and proteins) and technical challenges (e.g., peptide modifications). In addition, it is worth mentioning that genes with low mRNA-protein correlation often participate in the formation of large protein complexes, such as those related to complement activation, oxidative phosphorylation, and transcription initiation [74], resulting protein quantification that is less correlated with RNA measurements. Targets that could not be detected with the PRM method are not necessarily absent. Proteins and mRNA molecules are structurally and functionally different. Both molecules differ in the half-life rate, turnover, and stability [75]. In addition, some mRNAs may be translated into low abundance proteins, which could be below the detection limit of our proteomic assay [76]. Of note, the proteins that correspond to the mRNAs detected at the 24 h time point are not necessarily detectable at same time point. Thus, transcripts and proteins are temporally different, and some findings may derive from secondary responses to androgen, after the 24 h period. We should also consider the features of the various techniques used to detect and identify of mRNAs and proteins. The sensitivity and limit of detection of transcriptomic and proteomic techniques are different. Usually, transcriptomics is more sensitive than proteomics. Many transcripts do not show protein evidence or translated proteins may exhibit incomplete or wrong annotations of genes [77], explaining the absence of proteins that correspond to specific mRNAs.

Nevertheless, targeted mass spectrometry-based approaches are widely used in research for protein detection and quantification. The novel targeted MS-based approach of heavy-peptide labelled PRM shows high analytical performance, high sensitivity and high mass accuracy [78, 79]. This may be a useful tool for further implementation in more relevant systems [80], such as in cell line in vivo models, and patient-derived xenograft models [81, 82], with potential to be ultimately used in clinical assays. Thus, precision medicine can benefit from this approach by identifying biomarkers for monitoring disease progression or response to treatment, based on the patient’s proteome [81, 82].

To summarize, in BT474, DHT increases expression of cell proliferation-related genes, FAM genes related to the lipid synthesis and oxidation [58], which may be associated with decreased cell growth and increased cell differentiation [59]. On the other hand, we observed that FAM-related genes were also expressed in DHT-treated MDA-MB-453 cells, as previously observed by others [58], were associated with malignant phenotype in TNBC [73], and with increased cell proliferation [20, 73]. Taken this information into consideration, we speculate that AR can take advantage of the increased FAM to arrest tumor growth in luminal-type BCa, or to promote tumor progression in TNBC. Furthermore, knowing that MCF7 cells show increased proliferation, whereas T47D and ZR75.1 cells decreased [20], the differences in cell proliferation rate of androgen-treated BCa cells could be a result of the AR to ER ratio [5]. More specifically, AR causes increased tumor growth in TNBC with high AR levels (MDA-MB-453) [5, 83] and in ER^+^BCa with low AR levels (MCF7) [5, 84, 85]. On the other hand, AR acts as tumor suppressor in ER^+^BCa with high AR levels (BT474, T47D and ZR75.1) [20]. Nevertheless, this speculation needs further investigation.

Interaction analysis of these proteins revealed that AR is a significant regulator of gene expression in AR^+^ BCa, involving cross-talk with primary BCa-related genes that in turn activate secondary signaling pathways associated with BCa (reviewed in Ref. [69]). We can assume that DHT acts as a suppressor of cell proliferation and of anti-apoptotic processes, essential for cancer progression (Fig. 6). However, future extended inhibitory studies of AR and its partners will be very important to define the actual signaling action of AR and complete the identified primary targets (activator and repressor transcription factors), and subsequent AR-related secondary responses.

## Conclusions

The AR mechanism of action shows diverse effects in different BCa subtypes, indicating the necessity of more profound investigation of AR-mediated mechanism in a BCa subtype-based approach, which can be extended in therapies co-targeting the AR signaling along with other pathways (e.g., fatty acid metabolism) depending on the hormonal profiling of BCa. This information could be used in the treatment of BCa through the AR pathway that may act as a mechanism of tumor suppression by downregulating cancer-promoting pathways and preventing cell growth, in a personalized manner, that needs to be further elucidated.

## Supplementary Information

**Additional file 1. **Additional Tables S1–S13.

**Additional file 2. **Additional figures S1–S7.

**Additional file 3. **Additional Tables S1-S6.

## Data Availability

The datasets used and/or analyzed during the current study are included in this published article and its additional files. Any data not included in this article, are available from the corresponding author. The RNA sequencing raw and processed transcriptomic data have been deposited to Gene Expression Omnibus genomics data repository (https://www.ncbi.nlm.nih.gov/geo/) with the accession number GSE197321.

## Abbreviations

A: Androgen
AKT: Serine/threonine-protein kinase
AR: Androgen receptor
BCa: Breast cancer
DEGs: Differentially expressed genes
DHT: Dihydrotestosterone
ER: Estrogen receptor
FAM: Fatty acid metabolism
FC: Fold change
FDR: False detection rate
FPKM: Fragments per kilobase of exon per million reads mapped
HER2: Human epidermal growth factor receptor 2
LBD: Ligand binding domain
lncRNA: Long non-coding RNA
MYC: Avian myelocytomatosis viral oncogene homolog
ncRNA: Non-coding RNA
PD: Proteome discover
PI3K: Phosphoinositide 3-kinase
PR/ PGR: Progesterone receptor
PRM: Parallel reaction monitoring
PROG: Progesterone
PSA: Prostate specific antigen
RPKM: Reads per kilobase of transcript per million mapped reads
TF: Transcription factor
TFA: Trifluoroacetic acid
TNBC: Triple negative breast cancer
