# Supplementary material for: Transcriptome profiling and proteomic validation reveals targets of the androgen receptor signaling in the BT-474 breast cancer cell line
Source: Clin Proteomics. 2022 May 14;19:14. doi: 10.1186/s12014-022-09352-2 (PMC9107748; doi:10.1186/s12014-022-09352-2)
Supplement: Supplementary file 1 — Additional file 1. Additional Tables S1–S13. [file 12014_2022_9352_MOESM1_ESM.docx]

**Additional file 1**

**Table S1.** List of BCa cell lines that have been used to investigate the expression of the A-regulated candidate genes. The symbol “+/-” indicates the presence/absence of the specific receptor (AR: androgen receptor, ER: estrogen receptor, PR: progesterone receptor, HER2: human epidermal growth factor receptor 2).

| **#** | **BCa Cell lines** | **AR** | **ER** | **PR** | **HER2** | **Culture medium** | **Reference** |
| --- | --- | --- | --- | --- | --- | --- | --- |
| **1** | **BT-474** | **++** | **+** | **+++** | **+** | phenol-free RPMI-1640 media suppl with 1mM glutamine, 1mM sodium pyruvate, 0.01 mg/mL human recombinant insulin, | AR-expressed cell line [1]  KLK3/2 expressed [2] |
| **2** | **ZR-75-1** | **+++** | **++** | **++** | **-** | phenol-free RPMI-1640 | AR-expressed cell line [136]  KLK3/2 not expressed [2]  AR reduce cell proliferation [3] |
| **3** | **T47D** | **+** | **+** | **+** | **-** | phenol-free RPMI-1640 suppl with 0.007 mg/mL human recombinant insulin | **AR^-^**ER^+^BCa [4]  AR levels equal with BT474 [1]  KLK3/2 expressed [2]  AR reduce cell proliferation [3] |
| **4** | **MCF-7** | **+** | **+** | **+** | **-** | phenol-free RPMI-1640 | AR-expressed cell line [1]  AR induced tamoxifen resistance [5]  ΑR induced cell proliferation [6][3]  Suppressed by aromatase inhibitors [7]  KLK3/2 not expressed [2]  Enzalutamide treatment based on AR:ER ratio [8] |
| **5** | **MDA-MB-453** | **++++** | **-** | **-** | **-** | phenol-free RPMI-1640 | Highest AR levels AR^+^TNBC [4][9]  AR increases cell proliferation [9][3]  KLK3/2 not expressed [2]  AR^+^HER2^+^ BCa 🡪Targeting AR, WNT and HER2 pathways suppressed cell growth [10][11]  Enzalutamide treatment based on AR:ER ratio [8] |
| **6** | **SkBr3** | **+** | **-** | **-** | **+** | phenol-free RPMI-1640 | AR^+^HER2^+^ BCa [11]  AR targeting inhibits cell growth [12] |
| **7** | **MDA-MB-468** | **-** | **-** | **-** | **+** | phenol-free RPMI-1640 media suppl with 1mM glutamine, 1mM sodium pyruvate | AR^-^TNBC [4]/ AR^+^TNBC [13] |

**Table S2.** List of primers used in qRT-PCR for each candidate gene.

| **#** | **Genes** | **Forward** | **Reverse** | **Product size (bp)** | **reference** |
| --- | --- | --- | --- | --- | --- |
| 1 | *ZBTB16* | GAGCTTCCTGATAACGAGGCTG | AGCCGCAAACTATCCAGGAACC | 107 | Origene  [14] |
| 2 | *UGT2B11* | GTGGGCATTCCATTGTTTTT | TTACTGGTTGATCATGTTGAATTC | 193 | [15] |
| 3 | *KLK3* | CGCAAGTTCACCCTCAGAAGGT | CTTGAGTCTTGGCCTGGTCAT | 299 | Origene |
| 4 | *UGT2B28* | GTAGGCATTCCATTGTTTTG | TTACTGGTTGATCATGTTGAATTA | 193 | [15] |
| 5 | *CYP4F8* | ATGGCAGAGATGAAGGTGGT | TGATGCAAAGGTAGGTGGGT | 176 | Primer3 tool |
| 6 | *RANBP3L* | AAAGAGTGCTGAACAAGGTCCTG | GCAAGATTCCAGTGCCTTGTGTC | 130 | Origene |
| 7 | *KLK2* | GGCTCTGGACAGGTGGTAAAGA | CGGTAATGCACCACCTTGGTGT | 151 (v1, v3) , 188 v2 | Origene |
| 8 | *ZPLD1* | GTGATGCCAACCTCCACAGTAG | GCCTTCCATTCAGTGCCAGATC | 144 | Origene |
| 9 | *MYBPC1* | CCTATGAGGTCCGCATCTTTGC | GTCGTGTCAGTGACAGAGTCCA | 133 | Origene |
| 10 | *HAAO* | CCTGAGACAGAATGTGGACGTG | CTTGTGTTCGCTCCCAGGCATA | 143, 325 | Origene |
| 11 | *FKBP5* | GCGAAGGAGAAGACCACGACAT | TAGGCTTCCCTGCCTCTCCAAA | 123 | Origene |
| 12 | *AZGP1* | AGGACAGCCAACTTCAGAAGGC | CGATCTCACAACCAAACCTTCCC | 123 | Origene |
| 13 | *CYP4F3* | CCACCTACATCAAGCCTGTGCT | GGCTCCACTTTTCACCAGCACT | 151, 126 | Origene |
| 14 | *ALOX15B* | CAATGCCGAGTTCTCCTTCCATG | TGATGTGCAGGGTGTATCGGGT | 155 | Origene |
| 15 | *NABP1* | GCAGCATCACTATTTCCGTGTGG | GTGTCAGACATCCTTTCCACATG | 108 | Origene |
| 16 | *CXCL12* | CTCAACACTCCAAACTGTGCCC | CTCCAGGTACTCCTGAATCCAC | 105 | Origene |
| 17 | *PGR* | GTCGCCTTAGAAAGTGCTGTCAG | GCTTGGCTTTCATTTGGAACGCC | 136 | Origene |
| 18 | *WNT6* | GGTTATGGACCCTACCAGCA | AACTGGAACTGGCACTCTCG | 141 | [16] |
| 19 | *DKK1* | GGTATTCCAGAAGAACCACCTTG | CTTGGACCAGAAGTGTCTAGCAC | 125 | Origene |
| 20 | *GCSAM* | TCAGAGGAGCTGTGCTATACCC | GTCTCAGTTCCTCCCAAGGACT | 137 | Origene |
| 21 | *DKK2* | GGATGGCAGAATCTAGGAAGACC | CTGATGGAGCACTGGTTTGCAG | 150 | Origene |
| 22 | *S100P* | GATATTCGGGCAGCGAGGG | GCATCCTTGTCTTTTCCACTCTGC | 109 | [17] |
| 23 | *NDC80* | CTGACACAAAGTTTGAAGAAGAGG | TAAGGCTGCCACAATGTGAGGC | 128 | Origene |
| 24 | *ESR1* | GCTTACTGACCAACCTGGCAGA | GGATCTCTAGCCAGGCACATTC | 129 | Origene |
| 25 | *AR* | GAAGCCATTGAGCCAGGTGT | TCGTCCACGTGTAAGTTGCG | 164 | [18] |
| 26 | *HPRT1* | GCTGAGGATTTGGAAAGGGTGT | GGCCTCCCATCTCCTTCATCA | 94 | Sergi Clotet-Freixas |
| 27 | *GAPDH* | GTCTCCTCTGACTTCAACAGCG | ACCACCCTGTTGCTGTAGCCAA | 131 | Origene |

**Table S3.** Pathway resources included in the GSEA database. (Adopted from [19])

| **Source** | **File Origin** | **File Type** | **ID extracted** | **Frequency source is updated** | **Number of pathways** |
| --- | --- | --- | --- | --- | --- |
| KEGG [20] | KEGG ftp site (July 2011) | GMT | Symbol | static as of July 1, 2011 | 236 |
| Msigdb - c2 [21] | manual download | GMT | Entrez gene | sporadically | Biocarta - 217 Other - 47 |
| NCI [22] | scripted download of zipped release | BioPAX | Entrez gene | sporadically | 219 pathways |
| Institute of Bioinformatics (IOB) | received directly from IOB - static (July 2011) | BioPAX | Entrez gene | sporadically | 35 pathways - 10 are the same as CellMap, 1 is the same as NetPath |
| NetPath (IOB) [23] | scripted download of files numbered 1-25 | BioPAX | Entrez gene | static | 25 pathways - 12 are cancer pathways (10 are CellMap) 13 are immunity pathways |
| HumanCyc [24] | scripted download of zipped release | BioPAX | UniProt | updated periodically | 249 Pathways |
| Reactome [25] | scripted download of zipped release | BioPAX | UniProt | updated release | 1117 pathways (release 37) |
| GO [26] | scripted download from EBI ftp site | GAF | Uniprot | released once a month | 13,034 no GO IEA 15,181 with GO IEA |
| Msigdb - c3 [21] | manual download | GMT | Entrez gene | sporadically | 221 miRs 616 TFs |
| Panther [27] | scripted download of biopax archive | BioPAX | UniProt | updated periodically | 307 Pathways |

**Table S4.** Significant differentially expressed transcripts of the hormonally treated BT474 cells, identified by RNA sequencing. The experiment was performed in triplicates. DHT; dihydrotestosterone, PROG; progesterone, FC; fold change,

FDR p-value<0.05, red highlight represents the upregulated genes, blue color represents the downregulated genes, **bold** letters represent the commonly expressed genes in both conditions.

| **DHT-regulated genes** | | | | | **PROG-regulated genes** | | | |
| --- | --- | --- | --- | --- | --- | --- | --- | --- |
| **Gene name** | **FDR** | **log_2_ (FC)** | **FC** | | **Gene name** | **FDR** | **log_2_ (FC)** | **FC** |
| *ZBTB16* | 3.44E-85 | 10.18 | 1161.47 | | ***PGC*** | 2.22E-26 | 6.53 | 92.21 |
| *UGT2B11* | 2.44E-40 | 7.04 | 131.60 | | *KRT72* | 1.54E-04 | 5.84 | 57.22 |
| *KLK3* | 1.57E-05 | 6.29 | 78.19 | | *RBP2* | 8.71E-13 | 5.19 | 36.50 |
| *UGT2B28* | 4.67E-15 | 5.71 | 52.22 | | *KRT5* | 8.24E-39 | 5.08 | 33.83 |
| *CYP4F8* | 0.00E+00 | 5.61 | 48.81 | | ***OR2B6*** | 1.62E-03 | 4.93 | 30.47 |
| *RANBP3L* | 6.98E-47 | 5.22 | 37.37 | | *AFP* | 6.23E-03 | 4.65 | 25.11 |
| *KLK2* | 1.42E-02 | 4.66 | 25.33 | | *DDC* | 8.09E-77 | 4.62 | 24.67 |
| *ZPLD1* | 4.52E-02 | 4.49 | 22.53 | | *PXMP4* | 1.52E-02 | 4.49 | 22.42 |
| *MIR548D1* | 2.45E-02 | 4.49 | 22.50 | | *RIPPLY3* | 1.96E-02 | 4.30 | 19.75 |
| ***MYBPC1*** | 4.70E-62 | 4.49 | 22.48 | | *CALHM3* | 1.96E-02 | 4.30 | 19.75 |
| ***HAAO*** | 2.87E-10 | 4.44 | 21.71 | | *NME9* | 3.55E-02 | 4.09 | 17.08 |
| ***OR2B6*** | 2.72E-02 | 4.31 | 19.88 | | *C16orf3* | 3.55E-02 | 4.09 | 17.07 |
| *ESPNP* | 2.72E-02 | 4.31 | 19.88 | | *PASD1* | 3.55E-02 | 4.09 | 17.07 |
| *CASQ1* | 3.94E-11 | 4.22 | 18.66 | | ***LINC01226*** | 1.44E-04 | 3.91 | 15.05 |
| *C11orf91* | 4.96E-02 | 4.10 | 17.18 | | ***CALD1*** | 2.99E-24 | 3.08 | 8.44 |
| *TDRG1* | 4.96E-02 | 4.10 | 17.18 | | ***CXCL13*** | 1.87E-02 | 3.07 | 8.41 |
| *ADH1C* | 1.37E-03 | 4.07 | 16.80 | | ***MYBPC1*** | 3.35E-24 | 3.04 | 8.24 |
| *SEC14L2* | 0.00E+00 | 3.87 | 14.58 | | *LOC101927728* | 3.17E-02 | 2.94 | 7.67 |
| *RAB3B* | 2.36E-273 | 3.69 | 12.92 | | *PCDHA10* | 3.82E-02 | 2.94 | 7.67 |
| ***CTNNA2*** | 6.55E-21 | 3.61 | 12.23 | | ***PHGR1*** | 3.57E-03 | 2.90 | 7.45 |
| ***PGC*** | 2.49E-04 | 3.50 | 11.35 | | ***ARMC12*** | 9.26E-03 | 2.82 | 7.05 |
| *SLC26A3* | 3.43E-04 | 3.49 | 11.23 | | *GP2* | 5.61E-03 | 2.81 | 7.02 |
| *ATP1A4* | 5.25E-46 | 3.43 | 10.76 | | *AKR1B10* | 2.71E-56 | 2.69 | 6.44 |
| ***LINC01226*** | 5.80E-03 | 3.42 | 10.70 | | *ADAMTS18* | 1.56E-02 | 2.62 | 6.16 |
| *AKR1C3* | 2.25E-05 | 3.21 | 9.23 | | *LINC01134* | 1.56E-02 | 2.62 | 6.16 |
| ***ARMC12*** | 2.33E-03 | 3.13 | 8.78 | | ***HAAO*** | 1.96E-02 | 2.62 | 6.15 |
| *ZNF385B* | 4.24E-15 | 3.11 | 8.65 | | ***MME*** | 6.35E-39 | 2.51 | 5.69 |
| ***CXCL13*** | 2.58E-02 | 3.08 | 8.46 | | *PLB1* | 2.48E-04 | 2.46 | 5.49 |
| *UGT2B10* | 1.20E-04 | 3.04 | 8.25 | | ***PNMT*** | 2.28E-64 | 2.42 | 5.34 |
| *MINOS1-NBL1* | 4.37E-02 | 2.95 | 7.72 | | *KRT1* | 1.07E-26 | 2.36 | 5.12 |
| *DRD2* | 9.02E-03 | 2.91 | 7.50 | | *BMP3* | 6.35E-03 | 2.31 | 4.97 |
| *LBH* | 7.58E-09 | 2.86 | 7.28 | | *CACNA2D3* | 8.90E-03 | 2.20 | 4.60 |
| *GPM6A* | 1.02E-04 | 2.83 | 7.13 | | ***CTNNA2*** | 8.26E-05 | 2.12 | 4.36 |
| ***PHGR1*** | 1.40E-02 | 2.73 | 6.64 | | *LOC100134317* | 4.37E-02 | 2.12 | 4.34 |
| *IGF1* | 1.71E-13 | 2.71 | 6.53 | | *HIST1H1B* | 4.98E-02 | 2.02 | 4.04 |
| ***GJA1*** | 1.26E-07 | 2.65 | 6.28 | | *ACE2* | 2.44E-04 | 2.01 | 4.04 |
| *AZGP1P1* | 1.78E-07 | 2.65 | 6.27 | | *SULT1E1* | 4.27E-03 | 1.97 | 3.93 |
| *TUBA3E* | 3.66E-03 | 2.63 | 6.19 | | *CAPN6* | 1.05E-02 | 1.95 | 3.87 |
| ***FKBP5*** | 9.57E-23 | 2.61 | 6.10 | | *GSTA3* | 3.78E-02 | 1.94 | 3.85 |
| *C1orf116* | 0.00E+00 | 2.56 | 5.88 | | *SCNN1B* | 2.31E-09 | 1.93 | 3.80 |
| *NR0B2* | 3.86E-04 | 2.52 | 5.74 | | *GPX3* | 1.47E-02 | 1.91 | 3.77 |
| *TGM2* | 1.72E-08 | 2.49 | 5.63 | | *CST1* | 3.55E-18 | 1.90 | 3.73 |
| ***CALD1*** | 5.82E-11 | 2.30 | 4.93 | | *PKD1L2* | 8.23E-03 | 1.84 | 3.58 |
| *LOC102724596* | 4.18E-06 | 2.29 | 4.89 | | *LOC339874* | 2.67E-02 | 1.84 | 3.58 |
| ***AZGP1*** | 2.85E-22 | 2.28 | 4.84 | | *CNGA2* | 6.86E-04 | 1.78 | 3.43 |
| *MAOA* | 8.75E-14 | 2.26 | 4.81 | | *KYNU* | 6.01E-19 | 1.76 | 3.39 |
| *SLC15A5* | 3.63E-02 | 2.25 | 4.76 | | ***TSPAN7*** | 3.11E-08 | 1.76 | 3.39 |
| *TEX22* | 3.88E-02 | 2.22 | 4.67 | | *CD36* | 2.00E-03 | 1.73 | 3.33 |
| ***CYP4F3*** | 7.33E-33 | 2.22 | 4.66 | | *LOC101060553* | 3.88E-02 | 1.70 | 3.26 |
| *STEAP2* | 4.78E-02 | 2.21 | 4.62 | | *CDH5* | 4.15E-03 | 1.68 | 3.21 |
| *PLA2G5* | 2.26E-06 | 2.19 | 4.56 | | *DEPDC1-AS1* | 8.15E-05 | 1.67 | 3.19 |
| *TAT* | 2.01E-03 | 2.09 | 4.25 | | *LINC01121* | 3.19E-02 | 1.62 | 3.08 |
| *LRRC63* | 2.83E-03 | 2.08 | 4.22 | | *FAM178B* | 1.56E-16 | 1.60 | 3.03 |
| ***TSPAN7*** | 2.04E-11 | 2.04 | 4.11 | | *AKR1B1* | 3.16E-04 | 1.60 | 3.03 |
| *PCDH20* | 2.29E-36 | 2.03 | 4.08 | | *FMO1* | 3.59E-07 | 1.58 | 2.99 |
| ***UGT2B17*** | 5.31E-11 | 1.98 | 3.94 | | *ALDH1A2* | 2.01E-02 | 1.57 | 2.98 |
| *DNASE2B* | 3.87E-17 | 1.96 | 3.90 | | ***AZGP1*** | 1.01E-09 | 1.57 | 2.98 |
| *HRH3* | 3.71E-02 | 1.96 | 3.90 | | *S100P* | 3.30E-30 | 1.55 | 2.92 |
| ***PNMT*** | 1.10E-40 | 1.91 | 3.75 | | *DNAH12* | 2.15E-02 | 1.53 | 2.89 |
| ***ADCY2*** | 3.58E-16 | 1.90 | 3.73 | | *CYP2C9* | 1.18E-02 | 1.52 | 2.87 |
| *NBPF13P* | 2.65E-05 | 1.80 | 3.48 | | *CST4* | 5.65E-47 | 1.50 | 2.83 |
| *FLJ20021* | 3.45E-31 | 1.75 | 3.35 | | *HS3ST6* | 4.60E-05 | 1.49 | 2.81 |
| *CLDN8* | 7.31E-76 | 1.74 | 3.34 | | *MMP13* | 7.22E-11 | 1.42 | 2.68 |
| ***CYP4F12*** | 1.85E-45 | 1.73 | 3.32 | | *RDH5* | 1.97E-02 | 1.42 | 2.67 |
| *CYP2A6* | 2.32E-03 | 1.69 | 3.23 | | *INPP5D* | 5.22E-07 | 1.41 | 2.66 |
| *ALDH1A3* | 1.75E-31 | 1.68 | 3.20 | | *LINC01133* | 1.18E-10 | 1.39 | 2.63 |
| *SEMA6D* | 6.67E-05 | 1.67 | 3.19 | | *KRT2* | 5.73E-11 | 1.37 | 2.58 |
| *ASRGL1* | 6.26E-07 | 1.66 | 3.16 | | *ANKFN1* | 3.87E-03 | 1.36 | 2.56 |
| *UGT2B15* | 1.01E-10 | 1.66 | 3.16 | | *MYH15* | 3.12E-03 | 1.34 | 2.52 |
| *MYOZ1* | 4.80E-02 | 1.64 | 3.11 | | *A2ML1* | 2.95E-15 | 1.33 | 2.51 |
| *LINC00282* | 3.00E-02 | 1.64 | 3.11 | | *PIF1* | 6.11E-25 | 1.32 | 2.50 |
| *GNMT* | 7.54E-24 | 1.63 | 3.09 | | *SLC4A8* | 2.36E-07 | 1.30 | 2.46 |
| *CECR6* | 2.36E-14 | 1.63 | 3.09 | | ***CYP4F3*** | 5.08E-10 | 1.29 | 2.45 |
| *IQGAP2* | 1.76E-48 | 1.62 | 3.07 | | *LOC101930452* | 4.07E-02 | 1.29 | 2.44 |
| *ABCC12* | 3.44E-19 | 1.58 | 3.00 | | *LGALS1* | 2.88E-02 | 1.27 | 2.41 |
| ***OLR1*** | 1.06E-03 | 1.53 | 2.90 | | *BBOX1* | 2.57E-06 | 1.24 | 2.36 |
| *ISX* | 1.04E-03 | 1.52 | 2.87 | | *FAM72B* | 1.37E-21 | 1.23 | 2.35 |
| *NKX3-1* | 5.14E-47 | 1.52 | 2.86 | | *SNRPN* | 2.50E-03 | 1.23 | 2.34 |
| *DNAJC5B* | 3.99E-03 | 1.48 | 2.78 | | ***FKBP5*** | 1.14E-03 | 1.21 | 2.31 |
| *ALOX15B* | 1.44E-73 | 1.47 | 2.78 | | *MAMDC2* | 4.49E-03 | 1.21 | 2.31 |
| *SERHL2* | 4.98E-10 | 1.47 | 2.78 | | ***LAMA3*** | 2.04E-07 | 1.20 | 2.30 |
| *SYNPO2* | 2.97E-03 | 1.46 | 2.76 | | *SYNGR4* | 2.45E-04 | 1.19 | 2.29 |
| ***LAMA3*** | 4.73E-11 | 1.46 | 2.76 | | *BDKRB1* | 7.16E-06 | 1.18 | 2.26 |
| *MYLK3* | 9.78E-04 | 1.46 | 2.75 | | *HRASLS2* | 2.15E-09 | 1.18 | 2.26 |
| *PID1* | 2.33E-02 | 1.44 | 2.72 | | *FBXO43* | 1.18E-02 | 1.18 | 2.26 |
| *LDB3* | 2.60E-08 | 1.44 | 2.72 | | *LYN* | 1.50E-03 | 1.17 | 2.25 |
| *ANKK1* | 1.98E-12 | 1.44 | 2.71 | | *CDKN2C* | 4.42E-33 | 1.17 | 2.25 |
| *EAF2* | 1.73E-49 | 1.43 | 2.70 | | *DLGAP5* | 8.67E-46 | 1.15 | 2.22 |
| *SAMD12-AS1* | 1.07E-03 | 1.43 | 2.70 | | *KPNA7* | 2.97E-06 | 1.14 | 2.21 |
| *ARHGAP6* | 8.61E-13 | 1.42 | 2.68 | | *PTGS1* | 1.11E-08 | 1.14 | 2.21 |
| *SLC15A2* | 6.60E-28 | 1.41 | 2.67 | | *ALDH1L2* | 1.64E-02 | 1.14 | 2.21 |
| *PDK4* | 1.76E-08 | 1.40 | 2.63 | | *GSDMC* | 4.92E-02 | 1.13 | 2.19 |
| *KCNG3* | 1.08E-03 | 1.39 | 2.61 | | *PLEKHS1* | 3.19E-04 | 1.13 | 2.19 |
| *PTGES3L* | 2.16E-02 | 1.38 | 2.60 | | *CENPA* | 2.22E-26 | 1.13 | 2.18 |
| *PMEPA1* | 1.04E-124 | 1.37 | 2.58 | | *HMMR* | 3.00E-46 | 1.13 | 2.18 |
| *CROT* | 5.82E-71 | 1.37 | 2.58 | | *ENDOU* | 1.18E-02 | 1.12 | 2.17 |
| *TARP* | 5.90E-58 | 1.35 | 2.55 | | *DNAH6* | 2.54E-02 | 1.11 | 2.16 |
| *PIP* | 3.67E-24 | 1.33 | 2.51 | | *TINAGL1* | 1.92E-02 | 1.10 | 2.15 |
| *PLA2G2A* | 1.67E-21 | 1.33 | 2.51 | | *FAM13A-AS1* | 3.24E-02 | 1.10 | 2.15 |
| ***MME*** | 2.33E-09 | 1.32 | 2.50 | | *CDKN2D* | 1.20E-23 | 1.10 | 2.14 |
| *SLC11A1* | 4.56E-02 | 1.32 | 2.50 | | *NEURL1B* | 6.08E-45 | 1.09 | 2.13 |
| *LINC00930* | 1.54E-06 | 1.32 | 2.50 | | *MIRLET7BHG* | 2.89E-03 | 1.09 | 2.13 |
| *TTLL6* | 4.37E-02 | 1.31 | 2.49 | | *LINC00920* | 2.55E-03 | 1.09 | 2.13 |
| *SLC2A12* | 8.19E-05 | 1.31 | 2.48 | | *TMPRSS4* | 1.71E-06 | 1.08 | 2.12 |
| *SYNPO2L* | 6.29E-44 | 1.30 | 2.47 | | *PTTG1* | 2.62E-40 | 1.08 | 2.11 |
| *LAMC2* | 9.63E-15 | 1.27 | 2.42 | | *TMEM91* | 1.35E-02 | 1.08 | 2.11 |
| ***KLK11*** | 1.70E-02 | 1.27 | 2.41 | | ***CYP4F12*** | 4.88E-16 | 1.08 | 2.11 |
| *PSCA* | 4.94E-31 | 1.24 | 2.37 | | *RPGRIP1* | 1.16E-05 | 1.07 | 2.11 |
| *ACSM1* | 4.05E-06 | 1.23 | 2.34 | | *NDC80* | 2.72E-38 | 1.07 | 2.10 |
| *BANK1* | 7.07E-06 | 1.21 | 2.31 | | *VIPR2* | 1.42E-14 | 1.05 | 2.08 |
| *TP53TG1* | 5.71E-44 | 1.19 | 2.28 | | *MYLK2* | 8.15E-05 | 1.05 | 2.07 |
| *FAM209B* | 3.02E-04 | 1.18 | 2.27 | | *ANP32E* | 1.26E-58 | 1.05 | 2.07 |
| *PHOSPHO1* | 1.60E-05 | 1.18 | 2.26 | | *TIAF1* | 2.60E-02 | 1.05 | 2.07 |
| *PPP3CA* | 1.47E-116 | 1.17 | 2.25 | | *PLK1* | 9.75E-52 | 1.04 | 2.06 |
| *ALCAM* | 3.37E-108 | 1.17 | 2.25 | | *CENPE* | 1.20E-37 | 1.04 | 2.06 |
| *TMTC2* | 2.18E-64 | 1.16 | 2.24 | | *YBX2* | 1.47E-21 | 1.04 | 2.05 |
| *CES5AP1* | 2.86E-03 | 1.16 | 2.23 | | *CENPF* | 1.41E-40 | 1.03 | 2.05 |
| *HPGD* | 1.17E-12 | 1.15 | 2.21 | | *CASP14* | 9.47E-07 | 1.03 | 2.04 |
| *CNTNAP2* | 3.45E-69 | 1.14 | 2.21 | | *PSRC1* | 3.59E-26 | 1.02 | 2.02 |
| *AQP3* | 1.19E-02 | 1.12 | 2.17 | | *KIF20A* | 2.17E-61 | 1.01 | 2.02 |
| *RARB* | 1.53E-08 | 1.11 | 2.16 | | *SOX2* | 9.51E-06 | 1.01 | 2.01 |
| *LRFN2* | 4.28E-10 | 1.10 | 2.15 | | *DEPDC1B* | 3.41E-23 | 1.00 | 2.00 |
| *ADRB1* | 5.00E-04 | 1.10 | 2.14 | | *SEPT5* | 1.10E-02 | -1.00 | -2.00 |
| *STON2* | 2.16E-45 | 1.10 | 2.14 | | *SEMA6A* | 7.34E-07 | -1.00 | -2.01 |
| ***KLK12*** | 2.67E-03 | 1.09 | 2.13 | | *MATN3* | 2.07E-02 | -1.01 | -2.01 |
| *SCGB2A1* | 6.93E-06 | 1.09 | 2.12 | | *VSTM2L* | 1.63E-06 | -1.01 | -2.01 |
| *ABCC11* | 3.47E-30 | 1.09 | 2.12 | | *HTRA1* | 1.22E-06 | -1.01 | -2.02 |
| *CA2* | 2.70E-16 | 1.09 | 2.12 | | *EPHA8* | 6.17E-03 | -1.01 | -2.02 |
| *ADRB2* | 1.05E-03 | 1.08 | 2.11 | | *FLT4* | 2.18E-03 | -1.02 | -2.02 |
| *TPD52* | 1.04E-71 | 1.06 | 2.08 | | *VIM* | 1.23E-16 | -1.02 | -2.02 |
| *BCL6* | 2.63E-37 | 1.04 | 2.05 | | ***AREG*** | 1.94E-03 | -1.02 | -2.03 |
| *EPGN* | 4.72E-03 | 1.00 | 2.00 | | *NECAB2* | 2.28E-02 | -1.04 | -2.05 |
| *PHLDB2* | 4.00E-06 | -1.01 | -2.01 | | *JAKMIP1* | 2.05E-02 | -1.04 | -2.05 |
| ***FAM184B*** | 4.45E-04 | -1.01 | -2.01 | | *KCNC1* | 3.50E-05 | -1.04 | -2.05 |
| *SUSD3* | 2.62E-26 | -1.01 | -2.02 | | *EDN1* | 1.55E-13 | -1.05 | -2.06 |
| *FERMT1* | 1.29E-02 | -1.02 | -2.03 | | *ABAT* | 1.43E-59 | -1.05 | -2.07 |
| *NXPH1* | 9.66E-03 | -1.03 | -2.04 | | *NT5E* | 5.46E-03 | -1.05 | -2.07 |
| *MAP1A* | 4.78E-02 | -1.06 | -2.09 | | *COL12A1* | 4.75E-18 | -1.05 | -2.08 |
| *NABP1* | 7.77E-09 | -1.06 | -2.09 | | *RNF150* | 3.97E-03 | -1.06 | -2.08 |
| *FRMD3* | 1.32E-04 | -1.07 | -2.11 | | *RASGRP1* | 3.19E-05 | -1.08 | -2.11 |
| *PGBD5* | 7.86E-12 | -1.09 | -2.12 | | *ACE* | 2.21E-02 | -1.10 | -2.14 |
| *ANXA1* | 2.94E-29 | -1.11 | -2.16 | | ***PGR*** | 1.25E-39 | -1.11 | -2.16 |
| *UGT1A6* | 2.80E-04 | -1.12 | -2.17 | | *FOSB* | 4.96E-19 | -1.11 | -2.16 |
| *LOC101926943* | 3.68E-04 | -1.12 | -2.17 | | *TFF1* | 2.92E-74 | -1.12 | -2.17 |
| ***MAPK4*** | 7.48E-06 | -1.13 | -2.18 | | *C2orf82* | 2.04E-02 | -1.14 | -2.20 |
| *THRIL* | 1.82E-02 | -1.13 | -2.19 | | *MUM1L1* | 2.14E-17 | -1.14 | -2.20 |
| *PCED1B* | 6.87E-09 | -1.13 | -2.19 | | ***SYNDIG1*** | 2.08E-03 | -1.14 | -2.20 |
| *ST7-AS2* | 1.17E-02 | -1.14 | -2.20 | | *HIF1A-AS1* | 3.41E-02 | -1.14 | -2.21 |
| *CAV1* | 9.83E-04 | -1.14 | -2.21 | | *SOCS2-AS1* | 2.72E-04 | -1.15 | -2.23 |
| *KCNJ13* | 3.95E-05 | -1.15 | -2.22 | | *MTUS2* | 2.68E-02 | -1.15 | -2.23 |
| *RASSF2* | 4.26E-11 | -1.15 | -2.22 | | *EGR4* | 2.17E-05 | -1.17 | -2.24 |
| *NXNL2* | 7.24E-03 | -1.16 | -2.24 | | *GRM2* | 4.62E-02 | -1.17 | -2.25 |
| *LOC101929718* | 2.35E-02 | -1.17 | -2.24 | | *NPAS3* | 1.34E-09 | -1.18 | -2.26 |
| *ZRANB2-AS1* | 1.25E-02 | -1.17 | -2.26 | | *MGC45800* | 7.42E-05 | -1.19 | -2.29 |
| *CBLN2* | 1.59E-27 | -1.18 | -2.26 | | *SLIT1* | 6.12E-14 | -1.21 | -2.31 |
| *CCDC152* | 2.47E-02 | -1.20 | -2.29 | | ***KLK12*** | 4.12E-03 | -1.21 | -2.31 |
| ***NXPH3*** | 4.42E-05 | -1.20 | -2.30 | | ***WNT10B*** | 2.36E-02 | -1.21 | -2.32 |
| ***AREG*** | 3.30E-04 | -1.20 | -2.30 | | ***PTGER3*** | 1.28E-12 | -1.22 | -2.33 |
| *FBXO22-AS1* | 2.02E-02 | -1.20 | -2.30 | | *OLFML2A* | 4.76E-02 | -1.23 | -2.34 |
| *MCAM* | 1.94E-09 | -1.22 | -2.32 | | *AOX1* | 3.81E-03 | -1.24 | -2.35 |
| *MAP3K15* | 3.26E-03 | -1.23 | -2.35 | | *KCNH2* | 4.39E-04 | -1.24 | -2.36 |
| *SNHG22* | 1.60E-02 | -1.26 | -2.39 | | *RASD1* | 1.71E-06 | -1.24 | -2.37 |
| *LOC100129931* | 9.24E-03 | -1.27 | -2.41 | | *ZNF556* | 2.65E-02 | -1.25 | -2.38 |
| *ZNF488* | 2.08E-02 | -1.29 | -2.45 | | *MAML2* | 1.71E-03 | -1.26 | -2.39 |
| *SLC6A14* | 2.34E-11 | -1.31 | -2.48 | | ***MAPK4*** | 1.83E-07 | -1.28 | -2.43 |
| *CDH11* | 4.53E-03 | -1.31 | -2.48 | | *PLCL1* | 6.28E-04 | -1.29 | -2.44 |
| *DLC1* | 8.45E-07 | -1.31 | -2.48 | | *CCDC78* | 2.81E-20 | -1.29 | -2.45 |
| *SLC23A1* | 2.23E-02 | -1.31 | -2.49 | | *C1orf204* | 1.62E-03 | -1.30 | -2.47 |
| *AMIGO2* | 1.06E-35 | -1.32 | -2.50 | | *ARSJ* | 2.57E-16 | -1.31 | -2.47 |
| *NLGN4X* | 3.15E-10 | -1.32 | -2.50 | | ***ADCY2*** | 1.44E-03 | -1.31 | -2.48 |
| *SHROOM4* | 5.99E-06 | -1.33 | -2.51 | | *MLLT4-AS1* | 3.78E-02 | -1.31 | -2.48 |
| ***KCNQ5*** | 1.26E-08 | -1.33 | -2.52 | | *MYO1G* | 8.96E-06 | -1.32 | -2.50 |
| ***GFRA1*** | 5.91E-37 | -1.34 | -2.53 | | *AFAP1L2* | 5.02E-03 | -1.36 | -2.56 |
| *LRG1* | 4.43E-02 | -1.34 | -2.53 | | *ADAMTS9* | 2.81E-02 | -1.36 | -2.56 |
| *BCL2* | 3.51E-35 | -1.36 | -2.57 | | *TGFBR3* | 7.20E-03 | -1.37 | -2.58 |
| ***WNT10B*** | 1.41E-02 | -1.37 | -2.58 | | *JAZF1* | 1.69E-05 | -1.38 | -2.61 |
| ***FAM65B*** | 1.39E-02 | -1.38 | -2.61 | | ***FAM65B*** | 9.26E-03 | -1.38 | -2.61 |
| *DAB2* | 2.92E-02 | -1.40 | -2.63 | | ***PGAM2*** | 3.42E-02 | -1.38 | -2.61 |
| *LGR6* | 2.08E-02 | -1.44 | -2.71 | | *DDIT4L* | 3.25E-02 | -1.39 | -2.62 |
| *MIR1282* | 3.23E-03 | -1.44 | -2.72 | | ***CXCL12*** | 1.50E-22 | -1.39 | -2.62 |
| ***LINC01016*** | 7.66E-07 | -1.46 | -2.74 | | *MAN1A1* | 3.51E-125 | -1.40 | -2.65 |
| ***CXCL12*** | 2.89E-24 | -1.46 | -2.75 | | ***PLCE1*** | 1.55E-23 | -1.43 | -2.69 |
| *KCNK5* | 4.77E-11 | -1.47 | -2.77 | | *HEG1* | 1.69E-06 | -1.43 | -2.69 |
| ***PTGER3*** | 2.58E-17 | -1.49 | -2.81 | | ***LINC01016*** | 8.21E-07 | -1.43 | -2.70 |
| ***PGR*** | 1.19E-72 | -1.52 | -2.87 | | *LOC101929221* | 2.62E-02 | -1.43 | -2.70 |
| ***ASCL1*** | 1.10E-03 | -1.53 | -2.88 | | *TFF2* | 2.24E-02 | -1.45 | -2.72 |
| *PCDH9* | 1.53E-04 | -1.55 | -2.93 | | *ZDBF2* | 7.75E-03 | -1.45 | -2.73 |
| *NKX2-3* | 4.21E-02 | -1.55 | -2.94 | | *SLC2A9* | 1.86E-02 | -1.45 | -2.73 |
| *MSMP* | 1.96E-02 | -1.56 | -2.94 | | *CALCR* | 3.82E-09 | -1.47 | -2.77 |
| *SLC5A2* | 1.60E-03 | -1.57 | -2.96 | | ***NXPH3*** | 2.19E-07 | -1.49 | -2.80 |
| ***PGAM2*** | 2.02E-02 | -1.59 | -3.02 | | ***KLK11*** | 8.37E-03 | -1.49 | -2.82 |
| *WNT6* | 1.14E-03 | -1.59 | -3.02 | | *AATK* | 1.06E-02 | -1.53 | -2.88 |
| ***ANKRD1*** | 7.20E-03 | -1.63 | -3.10 | | *RASAL3* | 1.68E-10 | -1.57 | -2.96 |
| *ST7-OT3* | 8.57E-03 | -1.64 | -3.12 | | *NPB* | 1.99E-02 | -1.57 | -2.97 |
| *AGPAT9* | 2.29E-15 | -1.66 | -3.15 | | ***C6orf7*** | 1.86E-06 | -1.62 | -3.07 |
| *HTR7* | 1.13E-05 | -1.68 | -3.21 | | *HOXD3* | 4.94E-02 | -1.65 | -3.13 |
| *MC3R* | 5.15E-04 | -1.69 | -3.23 | | *SLC47A1* | 2.51E-02 | -1.66 | -3.15 |
| *DKK1* | 5.02E-36 | -1.70 | -3.26 | | *SYT9* | 2.26E-04 | -1.66 | -3.16 |
| *NKX6-1* | 1.72E-02 | -1.73 | -3.31 | | ***UGT2B17*** | 2.24E-03 | -1.66 | -3.16 |
| *POU3F3* | 4.50E-02 | -1.75 | -3.36 | | *DOC2GP* | 1.12E-03 | -1.67 | -3.18 |
| ***PLCE1*** | 5.20E-33 | -1.75 | -3.37 | | *PCDHGB2* | 3.46E-02 | -1.72 | -3.29 |
| *CTTNBP2* | 2.56E-28 | -1.79 | -3.46 | | *PRSS54* | 4.81E-02 | -1.73 | -3.31 |
| *FAM135B* | 1.17E-02 | -1.86 | -3.64 | | *LOC400685* | 4.42E-02 | -1.73 | -3.31 |
| *CSPG4* | 1.89E-02 | -2.01 | -4.03 | | *EGR3* | 2.01E-32 | -1.75 | -3.36 |
| *PRR16* | 4.67E-02 | -2.03 | -4.09 | | ***ANKRD1*** | 2.38E-03 | -1.76 | -3.39 |
| *GTF3C2-AS1* | 1.97E-03 | -2.06 | -4.17 | | *DCX* | 1.11E-02 | -1.77 | -3.40 |
| ***SYNDIG1*** | 6.90E-09 | -2.13 | -4.38 | | ***KCNF1*** | 1.30E-03 | -1.83 | -3.56 |
| *GLIS1* | 1.25E-02 | -2.14 | -4.41 | | *TFPI2* | 6.39E-05 | -1.87 | -3.66 |
| *LRTM2* | 2.47E-03 | -2.22 | -4.64 | | ***OLR1*** | 2.11E-02 | -1.96 | -3.89 |
| *ADARB2* | 2.90E-02 | -2.41 | -5.30 | | *NCAM1* | 2.64E-03 | -1.97 | -3.91 |
| ***KCNF1*** | 4.41E-05 | -2.44 | -5.41 | | *KLHL23* | 5.03E-06 | -2.03 | -4.09 |
| *PADI3* | 2.93E-02 | -2.56 | -5.90 | | ***KCNQ5*** | 3.41E-17 | -2.03 | -4.10 |
| *LOC100288162* | 4.75E-04 | -2.57 | -5.93 | | *ADIPOQ* | 3.09E-03 | -2.07 | -4.21 |
| ***TMEM132E*** | 3.71E-04 | -2.66 | -6.32 | | ***TMEM132E*** | 2.03E-03 | -2.13 | -4.36 |
| ***C6orf7*** | 3.20E-13 | -2.84 | -7.16 | | *CR2* | 4.56E-03 | -2.15 | -4.44 |
| *SLC9C1* | 1.99E-02 | -3.14 | -8.79 | | ***FAM184B*** | 1.93E-13 | -2.19 | -4.55 |
| *RPS17* | 6.97E-03 | -3.45 | -10.91 | | ***DKK2*** | 1.01E-04 | -2.20 | -4.61 |
| *BCAT1* | 3.19E-02 | -4.28 | -19.37 | | *PCP4* | 6.95E-04 | -2.27 | -4.83 |
| *TMEM176B* | 3.19E-02 | -4.28 | -19.37 | | *LPPR1* | 1.81E-04 | -2.31 | -4.95 |
| *ALLC* | 3.19E-02 | -4.28 | -19.37 | | *KCNH1* | 9.25E-05 | -2.31 | -4.97 |
| *LOC102467147* | 3.19E-02 | -4.28 | -19.37 | | *PHACTR1* | 2.77E-02 | -2.33 | -5.04 |
| *MIR324* | 2.15E-02 | -4.46 | -21.97 | | *ACAP1* | 4.81E-02 | -2.35 | -5.11 |
| *GCSAM* | 1.82E-02 | -4.46 | -22.00 | | *FGFBP2* | 4.81E-02 | -2.35 | -5.11 |
| ***DKK2*** | 2.06E-09 | -4.81 | -27.95 | | ***GFRA1*** | 5.79E-99 | -2.40 | -5.27 |
|  |  |  |  | | *IFITM1* | 9.07E-03 | -2.42 | -5.34 |
|  |  |  |  | | ***ASCL1*** | 5.30E-07 | -2.44 | -5.42 |
|  |  |  |  | | *FPR3* | 3.28E-02 | -2.47 | -5.52 |
|  |  |  |  | | *ADD3-AS1* | 3.54E-03 | -2.57 | -5.92 |
|  |  |  |  | *KCNH7* | | 2.20E-02 | -2.57 | -5.94 |
|  |  |  |  | *LRRTM4* | | 3.68E-02 | -2.89 | -7.41 |
|  |  |  |  | *LOC100506125* | | 2.39E-02 | -3.02 | -8.12 |
|  |  |  |  | *LINC01116* | | 2.21E-03 | -3.20 | -9.22 |
|  |  |  |  | *ARL5C* | | 1.02E-02 | -3.26 | -9.55 |
|  |  |  |  | *IRGM* | | 3.94E-02 | -4.07 | -16.75 |
|  |  |  |  | *IRX1* | | 3.94E-02 | -4.07 | -16.75 |
|  |  |  |  | *PRKG1-AS1* | | 3.94E-02 | -4.07 | -16.75 |
|  |  |  |  | *ALOX12B* | | 3.06E-02 | -4.27 | -19.34 |
|  |  |  |  | *CASC21* | | 2.96E-02 | -4.45 | -21.86 |
|  |  |  |  | *ITGB3* | | 1.79E-02 | -4.46 | -21.94 |
|  |  |  |  | *C22orf24* | | 1.21E-02 | -4.46 | -22.00 |
|  |  |  |  | *LOC643355* | | 1.33E-02 | -4.46 | -22.01 |
|  |  |  |  | *SERPINA11* | | 2.69E-02 | -4.47 | -22.17 |
|  |  |  |  | ***GJA1*** | | 4.15E-03 | -4.77 | -27.25 |

**Table S5.** Peptide list used for the development of PRM experiment

| **Accession** | **Gene Name** | **Protein description** | **Peptide sequence** | **Light Precursor m/z, charges (+)** | **Heavy**  **Precursor m/z, charges (+)** |
| --- | --- | --- | --- | --- | --- |
| O15296 | ALOX15B | Arachidonate 15-Lipoxygenase Type B | GFLNQESSGIPSSLETR | 911.4525++ | 916.4566++ |
| P10275 | AR | Androgen receptor | ELHQFTFDLLIK | 501.9450+++ | 504.6164+++ |
| P25311 | AZGP1 | Alpha-2-Glycoprotein 1, Zinc-Binding | EIPAWVPFDPAAQITK | 891.9749++ | 895.9820++ |
| P25311 | AZGP1 | Alpha-2-Glycoprotein 1, Zinc-Binding | HVEDVPAFQALGSLNDLQFFR | 801.7412+++ | 805.0773+++ |
| P48061 | CXCL12 | C-X-C Motif Chemokine Ligand 12 | ILNTPNCALQIVAR | 791.9403++ | 796.9445++ |
| Q08477 | CYP4F3 | Cytochrome P450 4F3 | VVLGLTLLR | 492.3342++ | 497.3384++ |
| P98187 | CYP4F8 | Cytochrome P450 4F8 | TLTSQGVDDFLQAK | 761.8910++ | 765.8981++ |
| O94907 | DKK1 | Dickkopf-related protein 1 | CYCGEGLSCR | 631.2416++ | 636.2457++ |
| O94907 | DKK1 | Dickkopf-related protein 1 | NGICVSSDQNHFR | 511.9003+++ | 515.2364+++ |
| Q9UBU2 | DKK2 | Dickkopf-related protein 2 | SAGMYQGLAFGGSK | 687.3295++ | 691.3366++ |
| Q13451 | FKBP5 | FKBP Peptidyl-prolyl cis-trans isomerase 5 | DVAFTVGEGEDHDIPIGIDK | 709.6795+++ | 712.3509+++ |
| P46952 | HAAO | 3-hydroxyanthranilate 3,4-dioxygenase | LMHQEQLK | 342.8515+++ | 345.5229+++ |
| P20151 | KLK2 | Kallikrein Related Peptidase 2 | HNLFEPEDTGQR | 481.5601+++ | 484.8962+++ |
| P07288 | KLK3 | Kallikrein Related Peptidase 3 | LSEPAELTDAVK | 636.8377++ | 640.8448++ |
| Q00872 | MYBPC1 | myosin binding protein C1 | ILTPLTDQTVNLGK | 756.9352++ | 760.9423++ |
| Q96AH0 | NABP1 | Nucleic acid binding protein 1 | GCLTLYTGR | 520.7633++ | 525.7674++ |
| O14777 | NDC80 | Kinetochore protein nuclear division cycle 80 homolog | YSVADIER | 476.7404++ | 481.7445++ |
| P06401 | PGR | progesterone receptor | TQDQQSLSDVEGAYSR | 892.4083++ | 897.4124++ |
| P06401 | PGR | progesterone receptor | DDAYPLYSDFQPPALK | 920.4436++ | 924.4507++ |
| Q969W9 | PMEPA1 | Prostate Transmembrane Protein, Androgen Induced 1 | TIFDSDLMDSAR | 685.8165++ | 690.8206++ |
| Q969W9 | PMEPA1 | Prostate Transmembrane Protein, Androgen Induced 1 | LHHTHIAPLESAAIWSK | 637.6794+++ | 640.3508+++ |
| Q86VV4 | RANBP3L | Ran-binding protein 3-like | IDVITGEETEHNVLK | 566.2966+++ | 568.9680+++ |
| P25815 | S100P | S100 Calcium Binding Protein P | ELPGFLQSGK | 538.2928++ | 542.2999++ |
| P25815 | S100P | S100 Calcium Binding Protein P | YSGSEGSTQTLTK | 679.8253++ | 683.8324++ |
| O75310 | UGT2B11 | UDP glucuronosyltransferase family 2 member B11 | HSGGLIFPPSYIPIVMSK | 648.3535+++ | 651.0249+++ |
| Q9BY64 | UGT2B28 | UDP glucuronosyltransferase family 2 member B28 | HSGGLIFPPSYIPVVMSK | 643.6816+++ | 646.3530+++ |
| Q9Y6F9 | WNT6 | Wnt family member 6 | ALVQLHNNEAGR | 441.2372+++ | 444.5733+++ |
| Q05516 | ZBTB16 | Zinc Finger and BTB Domain Containing 16 | LGELAVGMK | 459.2599++ | 463.2670++ |

**Table S6.** Final list of peptides for PRM validation

| **A/A** | **Final PRM peptide list** | **Peptide sequence** | **Limit of detection (fmol/μl)** |
| --- | --- | --- | --- |
| 1 | ALOX15B | GFLNQESSGIPSSLETR | 5 |
| 2 | AZGP1 | EIPAWVPFDPAAQITK | 10 |
| 3 | CXCL12 | ILNTPNCALQIVAR | 5 |
| 4 | DKK1 | CYCGEGLSCR | 1 |
| 5 | KLK3 | LSEPAELTDAVK | 1 |
| 6 | NABP1 | GCLTLYTGR | 10 |
| 7 | NDC80 | YSVADIER | 1 |
| 8 | PGR | TQDQQSLSDVEGAYSR | 5 |
| 9 | S100P | ELPGFLQSGK | 0.33 |

**Table S7.** GO annotation on the GAD_DISEASE_CLASS category of the DEGs in DHT-treated BT474 cells, using the DAVID (2021 update) tool. *Benjamini-Hochberg FDR adjusted p-value ≤0.05

| Category | Term | Count (%) | Fold Enrichment | FDR |
| --- | --- | --- | --- | --- |
| GAD_DISEASE_CLASS | METABOLIC | 102 (47.7) | 1.54 | 1.01E-07 |
| GAD_DISEASE_CLASS | UNKNOWN | 39 (18.2) | 1.92 | 2.19E-04 |
| GAD_DISEASE_CLASS | OTHER | 37 (17.3) | 1.85 | 5.39E-04 |
| GAD_DISEASE_CLASS | NEUROLOGICAL | 56 (26.2) | 1.54 | 6.31E-04 |
| GAD_DISEASE_CLASS | PHARMACOGENOMIC | 51 (23.8) | 1.49 | 2.33E-03 |
| GAD_DISEASE_CLASS | CANCER | 57 (26.6) | 1.42 | 2.87E-03 |
| GAD_DISEASE_CLASS | RENAL | 29 (13.6) | 1.63 | 9.44E-03 |
| GAD_DISEASE_CLASS | PSYCH | 37 (17.3) | 1.49 | 1.12E-02 |
| GAD_DISEASE_CLASS | IMMUNE | 51 (23.8) | 1.36 | 1.31E-02 |
| GAD_DISEASE_CLASS | CHEMDEPENDENCY | 59 (27.6) | 1.29 | 1.91E-02 |
| GAD_DISEASE_CLASS | NORMALVARIATION | 12 (5.6) | 1.85 | 5.88E-02 |
| GAD_DISEASE_CLASS | HEMATOLOGICAL | 27 (12.6) | 1.40 | 6.32E-02 |

**Table S8.** KEGG signaling pathway analysis of DEGs in DHT-treated BT474 cells. *Benjamini-Hochberg FDR adjusted p-value ≤0.05

| # | Term | Count | % | Genes | Fold Enrichment | p-value * |
| --- | --- | --- | --- | --- | --- | --- |
| 1 | hsa04976:Bile secretion | 10 | 4.7 | UGT2B10, UGT2B11, CA2, UGT2B15, ATP1A4, UGT2B17, UGT2B28, ADCY2, NR0B2, UGT1A6 | 9.40 | 1.26E-04 |
| 2 | hsa00830:Retinol metabolism | 9 | 4.2 | UGT2B10, ALDH1A3, CYP2A6, UGT2B11, ADH1C, UGT2B15, UGT2B17, UGT2B28, UGT1A6 | 11.07 | 1.26E-04 |
| 3 | hsa00982:Drug metabolism - cytochrome P450 | 9 | 4.2 | UGT2B10, CYP2A6, UGT2B11, ADH1C, MAOA, UGT2B15, UGT2B17, UGT2B28, UGT1A6 | 10.45 | 1.31E-04 |
| 4 | hsa00053:Ascorbate and aldarate metabolism | 6 | 2.8 | UGT2B10, UGT2B11, UGT2B15, UGT2B17, UGT2B28, UGT1A6 | 16.72 | 1.25E-03 |
| 5 | hsa00980:Metabolism of xenobiotics by cytochrome P450 | 8 | 3.7 | UGT2B10, CYP2A6, UGT2B11, ADH1C, UGT2B15, UGT2B17, UGT2B28, UGT1A6 | 8.58 | 1.34E-03 |
| 6 | hsa05207:Chemical carcinogenesis - receptor activation | 12 | 5.6 | UGT2B10, UGT2B11, BCL6, UGT2B15, BCL2, UGT2B17, UGT2B28, PGR, ADRB1, ADCY2, ADRB2, UGT1A6 | 4.73 | 1.34E-03 |
| 7 | hsa00040:Pentose and glucuronate interconversions | 6 | 2.8 | UGT2B10, UGT2B11, UGT2B15, UGT2B17, UGT2B28, UGT1A6 | 14.76 | 1.34E-03 |
| 8 | hsa00140:Steroid hormone biosynthesis | 7 | 3.3 | UGT2B10, UGT2B11, UGT2B15, AKR1C3, UGT2B17, UGT2B28, UGT1A6 | 9.60 | 2.05E-03 |
| 9 | hsa00860:Porphyrin and chlorophyll metabolism | 6 | 2.8 | UGT2B10, UGT2B11, UGT2B15, UGT2B17, UGT2B28, UGT1A6 | 11.67 | 3.28E-03 |
| 10 | hsa05204:Chemical carcinogenesis - DNA adducts | 7 | 3.3 | UGT2B10, CYP2A6, UGT2B11, UGT2B15, UGT2B17, UGT2B28, UGT1A6 | 8.48 | 3.28E-03 |
| 11 | hsa00983:Drug metabolism - other enzymes | 7 | 3.3 | UGT2B10, CYP2A6, UGT2B11, UGT2B15, UGT2B17, UGT2B28, UGT1A6 | 7.32 | 6.73E-03 |
| 12 | hsa00590:Arachidonic acid metabolism | 6 | 2.8 | CYP4F3, PLA2G2A, AKR1C3, PLA2G5, CYP4F8, ALOX15B | 8.23 | 1.30E-02 |
| 13 | hsa05200: Pathways in cancer | 14 | 6.5 | WNT10B, ZBTB16, PTGER3, LAMA3, KLK3, ADCY2, LAMC2, IGF1, WNT6, CXCL12, BCL2, RARB, CTNNA2, NKX3-1 | 2.20 | 1.25E-01 |

**Table S9.** Significant differentially expressed genes from the leading-edge subset of the top 20 gene sets from the GSEA analysis of the DHT-treated cells compared to controls.

| Gene name | Fold change | # of gene sets within the top 20 | Main function |
| --- | --- | --- | --- |
| *FKBP5* | 6.10 | 1 | Intracellular trafficking of steroid receptors; promote AKT/AKT1activity |
| *AZGP1* | 4.84 | 1 | Stimulates lipid degradation |
| *CYP4F3* | 4.66 | 3 | fatty acid metabolism |
| *CYP4F12* | 3.32 | 2 | fatty acid metabolism |
| *CYP2A6* | 3.23 | 2 | coumarin 7-hydroxylase activity |
| *ALDH1A3* | 3.20 | 1 | NAD-dependent aldehyde dehydrogenase that catalyzes the formation of retinoic acid |
| *IQGAP2* | 3.07 | 1 | Binds to activated CDC42 and RAC1; associates with calmodulin |
| *NKX3-1* | 2.86 | 1 | Transcriptional repressor; important role in normal prostate development; acts as a tumor suppressor controlling prostate carcinogenesis |
| *ALOX15B* | 2.78 | 2, 3 | fatty acid metabolism |
| *PMEPA1* | 2.58 | 1 | Negative regulator of TGF-beta signaling; role in cell proliferation, differentiation, apoptosis, motility, extracellular matrix production and immunosuppression; down-regulation of the AR by enhancing ubiquitination and proteasome-mediated degradation |
| *CROT* | 2.58 | 2, 3 | fatty acid metabolism |
| *ACSM1* | 2.34 | 2 | fatty acid metabolism |
| *HPGD* | 2.21 | 1, 2, 3 | fatty acid metabolism |
| *TPD52* | 2.08 | 1 | Tumor protein D52; association with prostate cancer and lung squamous cell carcinoma; related pathways are clathrin-derived vesicle budding and vesicle-mediated transport |
| *ANXA1* | -2.16 | 4 | **Innate immune response:** anti-inflammatory activity; promotes chemotaxis of granulocytes and monocytes. **Adaptive immune response:** regulation of the differentiation and proliferation of activated T-cells; negatively regulates hormone exocytosis; role in phagocytosis by mediating the Ca^2+^-dependent interaction between phagosomes and the actin cytoskeleton |
| *BCL2* | -2.57 | 14 | Suppresses apoptosis in a variety of cell systems |
| *CXCL12* | -2.75 | 14 | Induction of a rapid and transient rise in the level of intracellular calcium ions and chemotaxis; stimulation of monocytes and T-lymphocytes migration; essential for B-cell lymphopoiesis |
| *PGR* | -2.87 | 5, 14 | Transcriptional activator or repressor |
| *WNT6* | -3.02 | 15 | Activation of the canonical Wnt receptor signaling pathway |
| *DKK1* | -3.26 | 15 | Antagonizes canonical Wnt signaling; anti-apoptotic activity |

**Table S10.** Additional fatty acid metabolism-related genes, which are not included in the leading-edge gene list after the GSEA analysis of the RNA-seq results in DHT-treated BT474 cells. (Fold change)

| Gene name | A-regulated | BCa-expressed | Reference | note |
| --- | --- | --- | --- | --- |
| *UGT2B11*  (131.6) | Yes | ER^+^PR^+^ BCa cell line | [28, 29] | Crosstalk between androgen and lipid signaling |
| *UGT2B28*  (52.22) | Yes | Yes | [29] | Crosstalk between androgen and lipid signaling |
| *ACSL3* (1.22) | Yes | Luminal and HER2 BCa | [30] | Fatty acid activation (thioesterification) before any fatty acid synthesis or oxidation process. |
| *FASN* (1.21) | Yes | Luminal and HER2 BCa | [30, 31, 32] | Fatty acid synthesis. Its activity is upregulated by EGF signaling pathway induced by MARK, PI3K and SREBP1 pathways. |
| *ACACA* (1.50) | Yes | Malignant BCa, HER2 and ER^+^PR^+^ BCa | [30, 33] | Fatty acid synthesis; promotes palmitic acid synthesis and inhibits apoptosis, essential for BCa cell survival. |
| *SLC6A14*  (-2.48) | Not Known | TNBC | [30] | uptake of glutamine |

**Table S11.** Comparison of the AR mRNA expression levels between our results (qRT-PCR) and literature results (RNA sequencing data from CCLE, Cancer Cell Line Encyclopedia 2020 The Broad Institute of MIT & Harvard, https://portals.broadinstitute.org/ccle/page?gene=AR,). *RPKM, reads per kilobase of transcript per million mapped reads).

| **Cell line** | **Relative concentration (2^-ΔΔct^)** | **Log_2_(RPKM)*** |
| --- | --- | --- |
| MDA-MB-453 | 2.07 | 4.77 |
| BT474 | 1.38 | 3.43 |
| Zr75.1 | 0.42 | 3.78 |
| T47D | 0.20 | 2.39 |
| MDA-MB-468 | 0.06 | -2.60 |
| MCF7 | 0.02 | 2.22 |
| SKBR3 | 0.01 | -6.82 |

**Table S12.** Validated list of genes determined by RNAseq (*not significant with qRT-PCR)

| **A/A** | **Gene name** | **RNAseq ratio** | **qPCR ratio** |
| --- | --- | --- | --- |
| 1 | *ZBTB16* | 1161.47 | 62762.08 |
| 2 | *UGT2B11* | 131.6 | 81.12 |
| 3 | *KLK3* | 78.19 | 3742.71 |
| 4 | *UGT2B28* | 52.22 | 622.24 |
| 5 | *CYP4F8* | 48.81 | 100.79 |
| 6 | *RANBP3L* | 37.37 | 20.60 |
| 7 | *KLK2* | 25.33 | 2351.55 |
| 8 | *MYBPC1* | 22.48 | 21.39 |
| 9 | *HAAO* | 21.71 | 3.12 |
| 10 | *FKBP5* | 6.1 | 8.22 |
| 11 | *AZGP1* | 4.84 | 5.04 |
| 12 | *CYP4F3* | 4.66 | 5.08 |
| 13 | *ALOX15B* | 2.78 | 3.65 |
| 14 | *S100P** | 1.48 | 1.37 |
| 15 | *AR* | -1.59 | -1.33 |
| 16 | *NABP1* | -2.09 | -1.81 |
| 17 | *CXCL12* | -2.75 | -2.98 |
| 18 | *PGR* | -2.87 | -2.27 |
| 19 | *DKK1* | -3.26 | -3.33 |
| 20 | *DKK2* | -27.95 | -12.24 |
| 21 | *WNT6* | -3.02 | -6.92 |
| 22 | *PMEPA1* | 2.58 | Not tested |
| 23 | *NDC80* | 1.04 * | -1.51* |

**Table S13.** Descriptive statistics for Pearson correlation analysis between the two PRM experiments.

| Pearson r | RNA vs protein (PRM-A) | RNA vs protein (PRM-B) |
| --- | --- | --- |
| R | 0.7410 | 0.6988 |
| 95% confidence interval | 0.1514 to 0.9417 | 0.06479 to 0.9309 |
| R squared | 0.5491 | 0.4884 |
|  |  |  |
| P value |  |  |
| P (two-tailed) | 0.0224 | 0.0362 |
| P value summary | * | * |
| Significant? (alpha = 0.05) | Yes | Yes |
|  |  |  |
| Number of xy pairs | 9 | 9 |
