# Supplementary material for: Transcriptome profiling and proteomic validation reveals targets of the androgen receptor signaling in the BT-474 breast cancer cell line
Source: Clin Proteomics. 2022 May 14;19:14. doi: 10.1186/s12014-022-09352-2 (PMC9107748; doi:10.1186/s12014-022-09352-2)
Supplement: Supplementary file 2 — Additional file 2. Additional figures S1–S7. [file 12014_2022_9352_MOESM2_ESM.docx]

**Additional file 2**

FIGURES

A)

A B C

*ETOH*

*DHT*

*PROGif*

*ETOH*

*DHT*

*PROG*

*ETOH*

*DHT*

*PROG*

KLK3
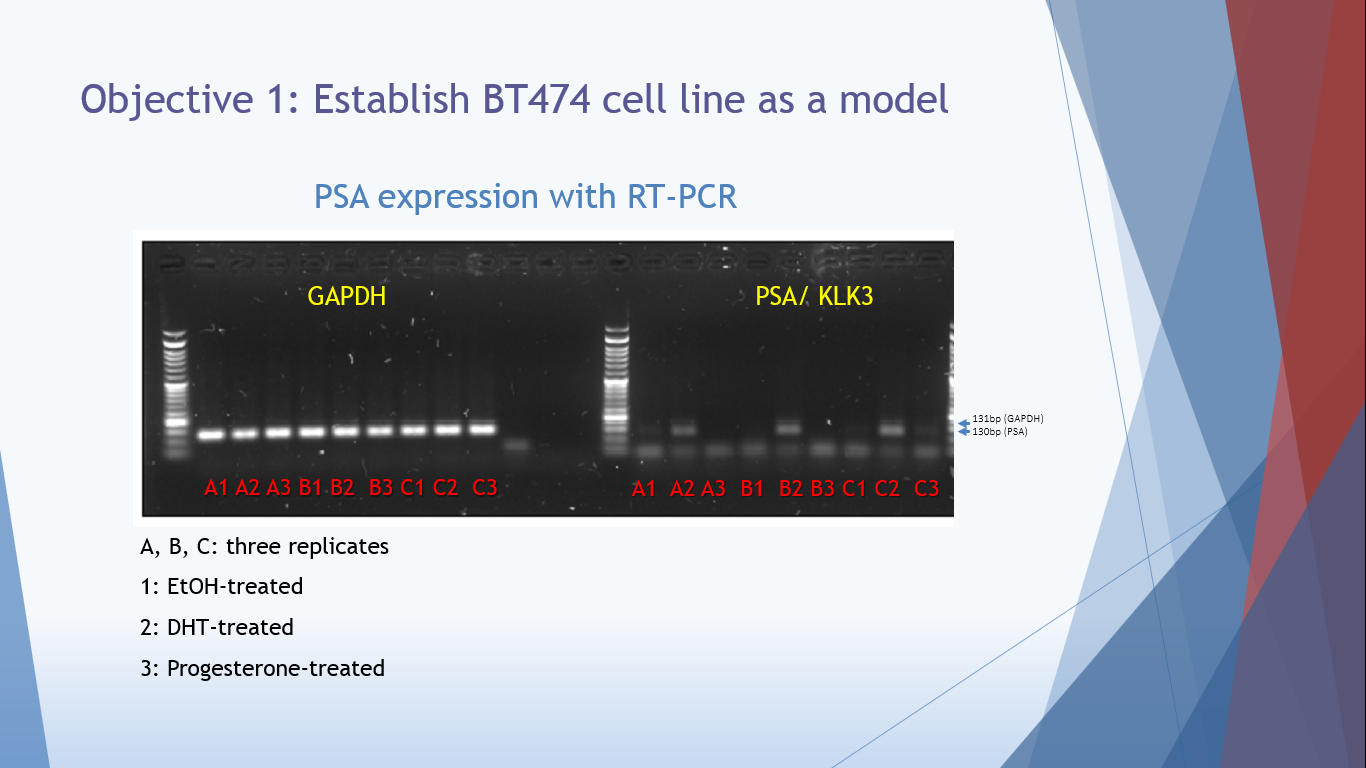


GAPDH
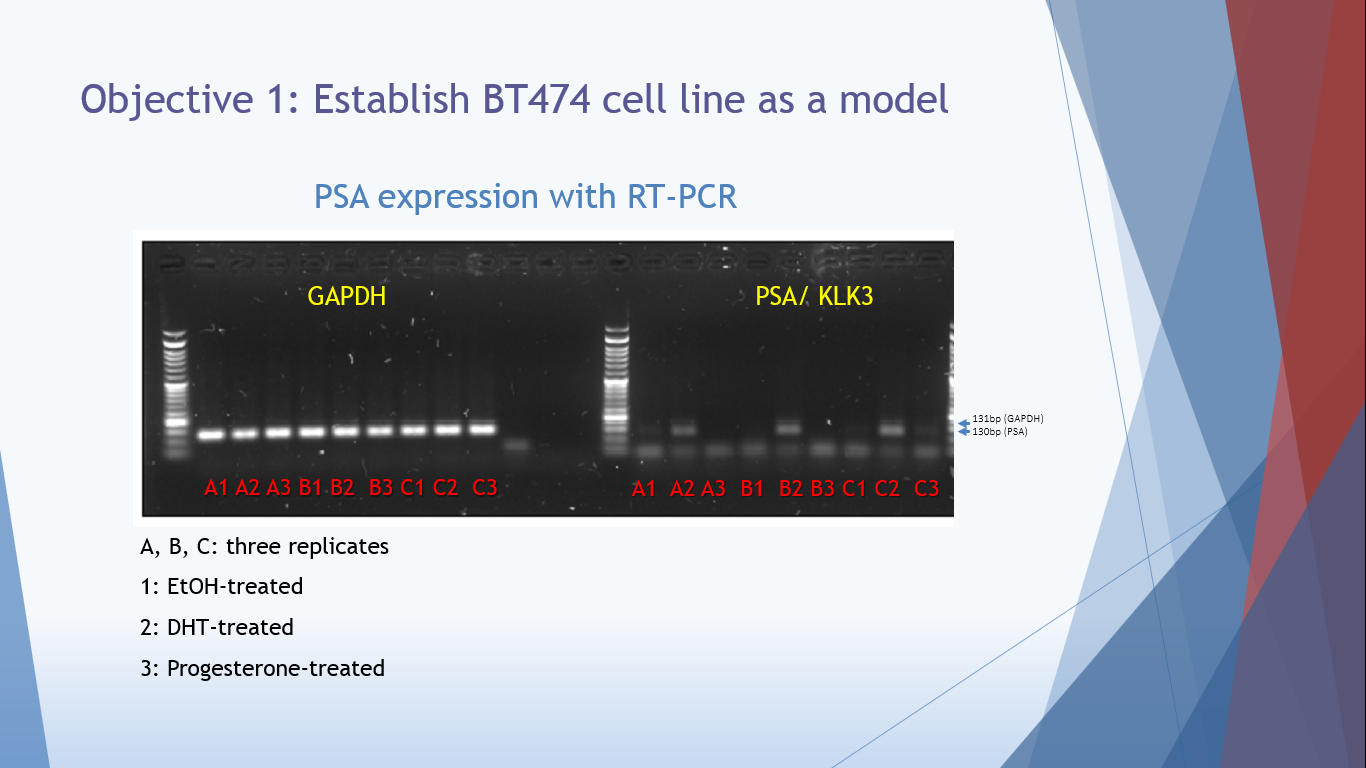


B)

**Figure S1. A)** KLK3 mRNA expression in BT474 cell line under various treatments. RT-PCR analysis of KLK3 mRNA shows that KLK3 is expressed specifically in DHT-treated cells. GAPDH gene was used to normalize gene expression. ETOH: ethanol (0.1%), DHT: dihydrotestosterone (10nM), PROG: progesterone (10nM), The experiment was performed in triplicates: A, B, and C. **B)** KLK3 protein expression levels in culture supernatants of BT474 cells during a 5-day stimulation with DHT (10nM). KLK3 levels (ng/L) were measured using ELISA and normalized based on the total protein concentration. The experiment was performed twice (A and B), using three replicates each time (student’s t- test; * p<0.05, ** p<0.01).

A) Molecular Function

B) Biological Process

C) Cellular Component

D)


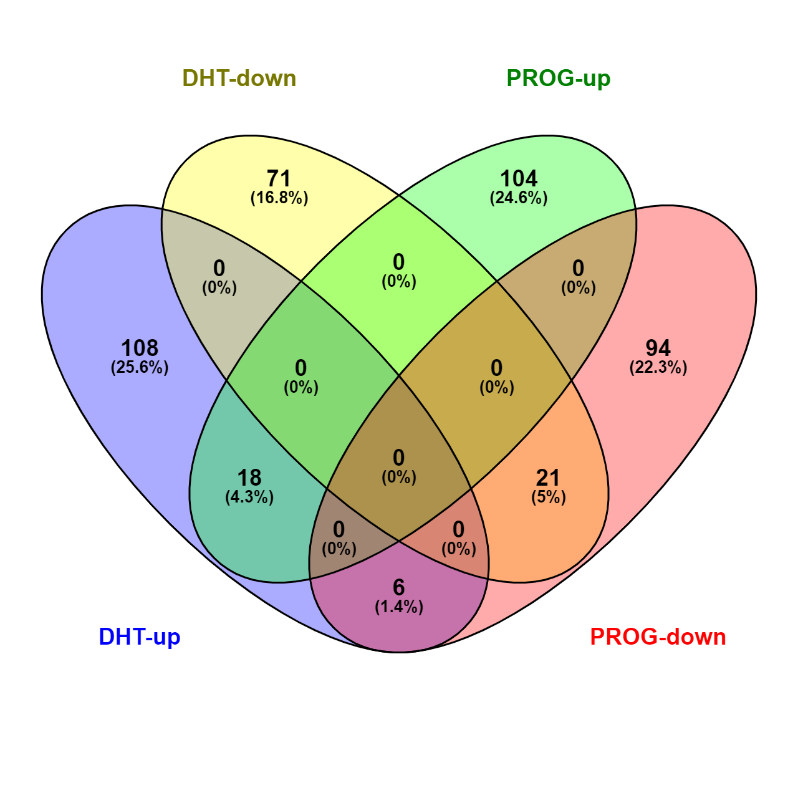


**Figure S2.** Gene Ontology (GO) and enriched pathway analysis of mRNA transcripts identified with RNAseq. The bar charts show the (A) molecular function, (B) biological process, and (C) cellular component analysis of differentially expressed protein-coding RNAs in the transcriptome of DHT-treated BT474 (blue) and PROG-treated BT474 (yellow) cells, after 24 hours of hormonal stimulation. D) Venn diagram representing the number and overlapping of DEGs in DHT and PROG-treated BT474 cells; DHT; dihydrotestosterone (10nM), PROG; progesterone (10nM).

A) B)

C)

ETOH DHT

***L*** A B A B ***L***

KLK2 (151bp)


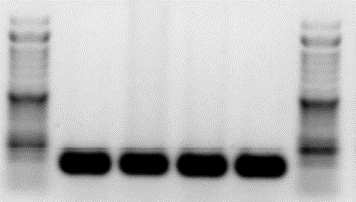


GAPDH (131bp)

ETOH DHT

***L*** A B C A B C ***L***

ZBTB16 (107bp)


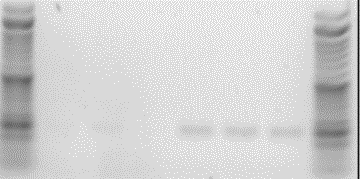


UGT2B11 (193bp)


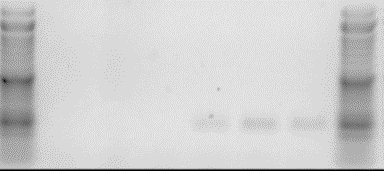


UGT2B28 (193bp)

AR (164bp)


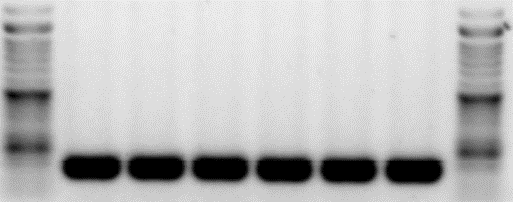


GAPDH (131bp)

**Figure S3.** Relative expression of selected genes that were validated using qRT-PCR in BT474 cells. A) All the genes show the same direction in expression with both techniques, RNA sequencing and qRT-PCR, except for *GCSAM*. The relative expression in qRT-PCR was calculated with the ΔΔCt method, normalized with GAPDH gene expression. The bars represent the fold change between DHT and control (ethanol)-treated BT474 cells. Statistical analysis was carried out with a false discovery rate (FDR) using a two-stage linear step-up procedure of Benjamini, Krieger and Yekutieli (GraphPad® Prism). Asterisks (*) next to gene name indicates non-significant genes. B) Relative expression of the two PROG-regulated genes, *NDC80* and *S100P*, used as negative controls in the DHT-related validation list. Only progesterone caused a significant increase in gene expression, compared with control and DHT-treatment. The relative expression was normalized with the housekeeping HPRT1 gene expression. Statistical analysis was carried out with a false discovery rate (FDR) using two-stage linear step-up procedure of Benjamini, Krieger and Yekutieli (GraphPad® Prism).*p-value <0.05; ns, non significant, p-value >0.05. C) Depiction of PCR products of representative validated genes that their FC expression could be visible on agarose gel. Biological replicates A, B, C; DNA ladder, ***L.***

A) B)

C)

**Figure S4.** Expression levels of AR-regulated genes during the inhibitory experiments of BT474 cells. A) The protein levels of the positive control gene, KLK3, were decreased after treatment with AR inhibitors. Genes with decreased (B) and increased (C) mRNA expression levels after AR inhibition. The protein levels were measured using ELISA, at 5 days post-treatment. The mRNA expression levels were measured by qRT-PCR in 24hr-treated BT474 cells. Treatments; 0.1% ethanol (control), 10nM DHT, 1000nM Enzalutamide and 10nM DHT, and 1000nM Flutamide and 10nM DHT. The experiment was performed in triplicate (student-t- test; *p<0.05, **p<0.01, *** p<0.001).

A)

B)

C)

D)

E)

**Figure S5.** (A) Expression levels of AR mRNA in various cell lines, using qRT-PCR. The relative AR concentration was calculated with the ΔΔCt method, normalized with the GAPDH gene expression. Samples were analyzed in triplicates. Expression of selected genes that validated using qRT-PCR in BCa cell lines. Genes of the validation list, as was defined from the RNA sequencing experiment. B) The expression profile of all seven BCa cell lines. C) The comparison of BT474 expression profile with the ER^+^PR^+^ BCa cell lines, Zr75.1, T47D, and MCF7. D) The comparison of BT474 expression profile with the ER^-^PR^-^ BCa cell lines, MDAMB453, SKBr3, and MDAMB468 cells. E) Comparison of BT474 expression profile with the high-expressing AR^+^ BCa cell lines, Zr75.1, and MDAMB453. The relative expression in qRT-PCR was calculated with the ΔΔCt method, normalized with the GAPDH gene expression. The bars represent the fold change between DHT and control (ethanol)-treated BT474 cells.

A)

Unknown pathway

Androgen-receptor pathway

Fatty acid metabolism

DNA recombination WNT-b pathway

ERBB4/ER-dependent pathway

B)

T47D Zr75.1 MCF7 MDA-MB-453 SKBr3 MDA-MB-468

***L*** E D E D E D E D E D E D ***L***


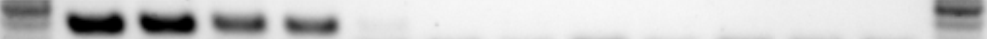


PGR - 136bp


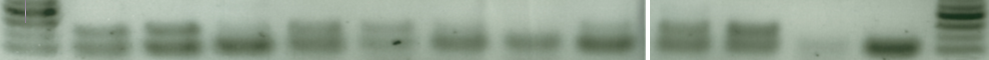


ZBTB16 - 107bp


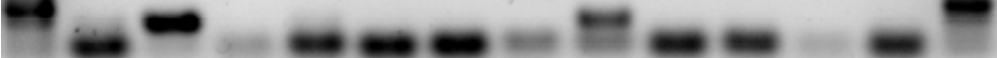


RANBP3L - 130bp


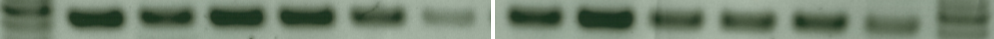


AR - 164bp


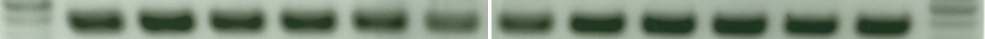


GAPDH - 131bp

**Figure S6.** Relative expression of representative genes from several molecular pathways that validated using qRT-PCR in BCa cell lines. A) The genes derived from the validation list, as was defined from the RNA sequencing experiment, and were classified based on the GSEA pathway analysis 3 as follows: Unknown pathway, Androgen-receptor pathway, Fatty acid metabolism, DNA recombination, ERBB4/ER-dependent pathway, and WNT-b pathway. The relative expression in qRT-PCR was calculated with the ΔΔCt method, normalized with the GAPDH gene expression. The bars represent the DHT (red color) and EtOH (control, black color)-treated BT474 cells. Statistical analysis was carried out with false discovery rate (FDR) using two-stage linear step-up procedure of Benjamini, Krieger and Yekutieli (GraphPad® Prism). B) PCR products of representative genes expressed on various BCa cell lines, run on agarose gel. ***L***, DNA ladder; E/ETOH, ethanol; D/DHT, dihydrotestosterone***.***

A)

B) C)

**Figure S7.** Expression of validated proteins (previously identified with RNA sequencing) using the targeted PRM approach. A) Proteins that show upregulation with DHT (10nM) stimulation of BT474 cells. B) Proteins that show downregulation after DHT-treatment of BT474 cells. C) Expression of representative validated proteins showing the intensity values as measured by PRM. KLK3 intensities were completely absent at the 24hr time-period and in all control (ethanol) samples. Only in DHT-treated cells, intensities were detected at the 5-day period. PGR intensity was almost absent in DHT-treated cells compared to control cells, especially after 5 days post-treatment. All measurements were performed in triplicates for proteome samples and in duplicates for secretome samples. *p<0.05; **p<0.01; ***p<0.001 (Student’s t-test).
